# There Are Multiple Clocks That Time Us: Cross-Sectional and Longitudinal Associations Among 14 Alternative Indicators of Age and Aging

**DOI:** 10.1093/gerona/glae244

**Published:** 2024-10-09

**Authors:** Johanna Drewelies, Jan Homann, Valentin Max Vetter, Sandra Düzel, Simone Kühn, Laura Deecke, Elisabeth Steinhagen-Thiessen, Philippe Jawinski, Sebastian Markett, Ulman Lindenberger, Christina M Lill, Lars Bertram, Ilja Demuth, Denis Gerstorf

**Affiliations:** Center for Environmental Neuroscience, Max Planck Institute for Human Development, Berlin, Germany; Center for Lifespan Psychology, Max Planck Institute for Human Development, Berlin, Germany; Division of Lipid Metabolism, Department of Endocrinology and Metabolic Diseases, Charité Universitätsmedizin Berlin, Berlin, Germany; Friede Springer Cardiovascular Prevention Center, Charité–Universitätsmedizin Berlin, Berlin, Germany; Center for Environmental Neuroscience, Max Planck Institute for Human Development, Berlin, Germany; Max Planck UCL Centre for Computational Psychiatry and Ageing Research, Berlin, Germany, and London, UK; Center for Lifespan Psychology, Max Planck Institute for Human Development, Berlin, Germany; Division of Lipid Metabolism, Department of Endocrinology and Metabolic Diseases, Charité Universitätsmedizin Berlin, Berlin, Germany; Department of Psychology, Humboldt Universität zu Berlin, Berlin, Germany; Department of Psychology, Humboldt Universität zu Berlin, Berlin, Germany; Center for Environmental Neuroscience, Max Planck Institute for Human Development, Berlin, Germany; Max Planck UCL Centre for Computational Psychiatry and Ageing Research, Berlin, Germany, and London, UK; Center for Lifespan Psychology, Max Planck Institute for Human Development, Berlin, Germany; Ageing Epidemiology Unit, School of Public Health, Imperial College London, London, UK; Lübeck Interdisciplinary Platform for Genome Analytics (LIGA), University of Lübeck, Lübeck, Germany; Division of Lipid Metabolism, Department of Endocrinology and Metabolic Diseases, Charité Universitätsmedizin Berlin, Berlin, Germany; Department of Psychology, Humboldt Universität zu Berlin, Berlin, Germany; German Institute for Economic Research, DIW Berlin, Berlin, Germany; (Biological Sciences Section)

**Keywords:** Age indicator, Aging, BASE-II, Biological age, Epigenetic clock

## Abstract

Aging is a complex process influenced by mechanisms operating at numerous levels of functioning. Multiple biomarkers of age have been identified, yet we know little about how the different alternative age indicators are intertwined. In the Berlin Aging Study II (*n*_min_ = 328; *n*_max_ = 1 517, women = 51%; 14.27 years of education), we examined how levels and 7-year changes in indicators derived from blood assays, magnetic resonance imaging brain scans, other-ratings, and self-reports converge among older adults. We included 8 epigenetic biomarkers (incl. 5 epigenetic “clocks”), a BioAge composite from clinical laboratory parameters, brain age, skin age, subjective age, subjective life expectancy, and subjective health horizon. We found moderate associations within aging domains, both cross-sectionally and longitudinally over 7 years. However, associations across different domains were infrequent and modest. Notably, participants with older BioAge had correspondingly older epigenetic ages. Our results suggest that different aging clocks are only loosely interconnected and that more specific measures are needed to differentiate healthy from unhealthy aging.

Aging encompasses multidimensional processes that exert diverse effects on various organs and tissues. It can be described as multifaceted processes characterized by the time-dependent decline of functional capacity and stress resistance, which is associated with an increased risk of morbidity and overall mortality (1–5). At the same time, one of the hallmarks of aging is its heterogeneity within a given population, resulting from often complex associations among a myriad of different factors including genetics, epigenetics, biology, individual behavior, and the environment. Due to this complexity, relying solely on chronological age fails to capture the diverse aging trajectories both between persons and within a given person. Previous work has highlighted the simultaneous operation of multiple clocks that are linked to (behavioral) change (5), that development unfolds at different paces at different levels of analysis, and that human development is co-constructed by multiple processes that each evolve at their own speed (bidirectional traffic (6,7)). Consistent with this overarching idea, the concept of “biological age” has emerged, aiming to better account for the variability and heterogeneity observed in aging processes (8,9). Biological age markers reflect an individual’s aging state relative to their chronological age. Given that aging affects nearly all tissues and organs of the body, research has identified various markers (also referred to as “clocks”) of aging (10), including epigenetic age, brain age, and other composite markers (eg, based on laboratory parameters). While each of these age indicators offers unique insights into an individual’s aging processes, only little is known about the interrelation between markers. However, this knowledge is crucial for gaining a comprehensive understanding of the underlying mechanisms of aging and guiding interventions to promote healthy aging and reduce age-related morbidities (11).

The field of biological aging research has witnessed significant advancements in recent years, with the development of novel biomarkers and technologies to assess aging processes. For example, Levine (12) suggested the use of 10 biomarkers encompassing multiple physiological systems to create a biological age score that outperforms chronological age in predicting mortality. Belsky et al. (13) pursued a distinct approach (see also (14)) and operationally defined among young adults the Pace of Aging as a longitudinal change phenotype that was then also found to be associated with functional decline, particularly cognitive ability. Furthermore, novel high-throughput molecular approaches now allow to interrogate large parts of an individual’s epigenome allowing the identification of “epigenetic clocks” based on DNA methylation (DNAm) patterns. To this end, Horvath (15) and Hannum et al. (16) utilized genome-wide DNAm data to develop age prediction models incorporating multiple DNAm sites. Furthermore, the concept of “age acceleration,” characterized by the difference between DNAm age and chronological age, has been linked to various mortality risk factors and has shown predictive utility for all-cause mortality (17–19).

Interestingly, previous empirical studies have indicated that different age indicators exhibit only weak to moderate correlations, and the nature of these associations can vary across populations and contexts (20). For instance, research by Belsky et al. (13,21) demonstrated both weak cross-sectional and longitudinal correlations between biomarkers of aging, such as telomere length (TL) and biomarker composite scores, suggesting that these indicators may capture distinct aspects of aging. Similarly, Robinson and colleagues (22) examined cross-sectional associations between metabolomic age acceleration and DNAm age acceleration (DNAmAA, Horvath and Hannum clocks) and found no correlation between the two. Pearce et al. (23) found only limited cross-sectional associations between relative leukocyte telomere length (rLTL) and epigenetic clocks indicating that both may capture different underlying biological mechanisms associated with cellular aging. Similar findings have also been reported from other studies (24,25). However, some age indicators, such as GrimAge and DunedinPACE (both based on DNAm patterns), have shown stronger correlations, reinforcing the need for careful consideration of the specific constructs and contexts when interpreting aging biomarkers (26).

It is also worth noting that certain associations between age indicators have been studied more extensively, whereas associations between other indicators remain largely unexplored. For example, several studies have investigated associations between indicators of aging developed from genetic, epigenetic, and clinical laboratory data (9,27). Similarly, biological aging has been linked to cognitive and physical aging. To illustrate, previous work using data from the Berlin Aging Study II (BASE-II) has linked Vitamin D status & supplementation with 7-CpG clock and Horvath clocks over time (28), 7-CpG DNAmAA epigenetic clock with cardiovascular health (29), PhenoAge and GrimAge clocks with metabolic syndrome (30), and 7-CpG clock with risk of diabetes complications (longitudinal analysis, (31)). The objective of this article is, therefore, not to demonstrate the validity of the alternative age markers. Instead, we are operating under the overarching assumption that the level of validation is good to very good for some biomarkers of aging (eg, for a comprehensive overview of the blood-based composite markers, see (32); for subjective age, see (33)), but less clear for others (see also a select overview Supplementary Table 1).

However, we are only beginning to understand how markers of biological age such as laboratory parameter composites and epigenetic age are associated with alternative markers of aging in other key areas of life. For example, it remains largely unclear whether and how a laboratory parameter composite marker indicating older biological age or epigenetic age relates to other-ratings of older skin age and/or subjective age. Also, the current body of research lacks a comprehensive examination of longitudinal changes across different age indicators and instead focuses only on selected indicators (for an exception see (32)). As a consequence, our understanding of the intricate interplay between different biological age indicators and the extent to which within-person age-related changes in one indicator are associated with those changes in other indicators remains very limited.

In this study, we aim to address the knowledge gap noted by examining 2 sets of research questions. In the first step, we investigate whether and how a large variety of 14 different indicators of biological age and aging converge and diverge within older adults. These indicators are derived from various sources: blood assays (including epigenetic clocks, pace of aging, and TL estimated from epigenetic data such as Dunedin PACE and DNAmTL), laboratory parameters (BioAge), magnetic resonance imaging (MRI) brain scans (brain age), other-ratings (skin age), and self-reports (subjective age, subjective life expectancy, and subjective health horizon).

Using these data, we conduct a comprehensive analysis of available biomarkers in BASE-II to examine how these are interrelated with one another. In a second step, we explore how these constellations change as people grow older and study associations between changes in these age indicators over the course of 7 years.

The findings from this study may help the development of better models of aging and help devise strategies for promoting healthy aging.

## Method

### Participants and Procedure

The BASE-II is a longitudinal study that aims to identify factors that promote healthy aging. Participants were recruited through advertisements in local newspapers and on public transport in the greater Berlin area, Germany. At baseline examination (2009–2014), 2 171 participants were medically examined (~75% aged 60–84 years and ~25% aged 20–37 years; the younger age group was not considered in the present work). In this study, we focus on the analysis of 1 083 BASE-II participants of the older age group who were reexamined on average 7.4 years after baseline as part of the GendAge project, which is a sub-study of the BASE-II. For more detailed cohort information at baseline and follow-up, please refer to, for example, Demuth et al. (34). Importantly, missingness was not equal across indicators of aging, which was mostly driven by the design of our large-scale study and available funding over time. To maximize the sample size, we thus decided to use pairwise deletion. In a series of robustness checks, we obtained results that are by and large consistent with those reported in the main text when we examined associations (i) among those who provided data to all variables under study (ie, using complete cases with listwise deletion, see Supplementary Figures 5 and 6), (ii) separately for women (see Supplementary Figure 7) and men (see Supplementary Figure 8), (iii) among those who suffered from 2 and more chronic physical diseases at follow-up (ie, excluding supposedly healthy participants; see Supplementary Figure 9), and (iv) among those who were reassessed within 6 years (ie, excluding those with long time intervals in between baseline and follow-up, see Supplementary Figure 10).

All participants gave written informed consent. The medical assessments at baseline and follow-up were conducted in accordance with the Declaration of Helsinki and approved by the Ethics Committee of the Charité – Universitätsmedizin Berlin (approval numbers EA2/029/09 and EA2/144/16). They were registered in the German Clinical Trials Registry as DRKS00009277 and DRKS00016157. The Ethics Committee of the Max Planck Institute for Human Development approved the procedure, and the Ethics Committee of the German Society for Psychology (DGPs) additionally approved the MRI protocol.

### Age Measures

*Chronological age* was calculated based on the year and month of the assessment/interview relative to the year and month of birth.

#### BioAge

To operationally define biological age (referred to as *BioAge*), we selected 12 standard blood laboratory parameters that were identified in the first Berlin Aging Study as predicting mortality (35). Specifically, measured in a clinical routine laboratory with appropriate quality standards, we selected parameters across metabolic, cardiovascular, inflammatory, and kidney functioning: zinc, sodium, chloride, uric acid, albumin, alpha-1 globulin, alpha-2 globulin, HbA1c, hemoglobin, leukocytes, lymphocytes, and creatinine (35).

#### Epigenetic biomarkers

Based on methylation data determined with the “Infinium MethylationEPIC” array (Illumina, Inc., San Diego, CA, USA), DNAm age was estimated using Horvath’s clock (15,36), Hannum’s clock (16) (often called the first generation clocks), DNAm PhenoAge (37), and GrimAge (38) (clocks of the second generation), and we additionally estimated DunedinPACE (26) (pace of aging, third generation clock) and DNAmTL (36) (TL). On the EPIC array only 512 of the original 513 CpG sites and 64 of the original 71 CpG sites were available for the estimation of DNAm PhenoAge and Hannum’s clock, respectively (39). We note that blood (leukocyte) DNAm data was available from 2 time points, baseline and follow-up, whereas the buccal swaps were taken only at follow-up. The Horvath algorithm among the epigenetic clock algorithms analyzed in our study is the only one trained for usage in multiple different tissues (“multi-tissue predictor”).

DNAm profiling was performed on the “Infinium MethylationEPIC” array (Illumina, Inc.) on aliquots of DNA extracts derived from blood at ~50 ng/µl concentration. Data preprocessing was performed with R 4.1.2 and the R-package “Bigmelon” using recommended default parameters for quality control (Gorrie-Stone et al., 2019). Briefly, probes were filtered according to the detection *p* value. Probes with more than 1% of samples having a detection *p* value of .05 were removed from the analysis, as well as probes with a bead count smaller than 3 in more than 5% of the samples. Then, outliers were identified with the outlyx function based on 2 times the interquartile range of the first PC of the DNAm data and the pcout function with a threshold of 0.15. Pcout is a function to identify outliers computationally fast in high-dimensional data (40). Additionally, samples with a bisulfite conversion efficiency below 80% as estimated by the bscon function were removed.

Based on these methylation data DNAm age (DNAm age) was estimated using Horvath’s clock (15,36), Hannum’s clock (16), DNAm PhenoAge (37) , and GrimAge (38) (via the Online DNA Methylation Age Calculator with use of the option “Normalize Data” (https://dnamage.genetics.ucla.edu/new), and we additionally estimated DunedinPACE (26) (pace of aging) and DNAmTL (36) (TL). The 7-CpG clock (another first generation clock) was determined from methylation data through methylation-sensitive single nucleotide primer extension (MS-SNuPE) from samples collected at baseline examination of the participants analyzed in this study (39). For a more detailed description of the methods used, see (41,42). DNAm age acceleration was calculated as the unstandardized residuals obtained from a linear regression analysis in which DNAm age was regressed on chronological age and leukocyte cell distribution (including neutrophils, monocytes, lymphocytes, and eosinophils measured in G/l). The measurement of blood cell composition was performed by an accredited clinical biochemistry laboratory (MVZ Labor 28 GmbH, Berlin, Germany) using automated standard methods, specifically flow cytometry. To account for the known relationship between blood cell–type composition and chronological age, we utilized a model adjusted for blood cell counts.

*Skin age* was estimated by quantifying lentigines on both hands from hand photographs taken of each BASE-II participant. Photos were taken at baseline assessment and examined independently by 3 raters in separate sessions. The amount and size of lentigines on the back of the hands were quantified using a 4-level categorical score (ranging from 0 = *no or very few lentigines*) to 3 = *very abundant presence of lentigines on both hands*). The resulting lentigines score per BASE-II participant represents the unit-weighted average of the score assigned to each photograph by the 3 raters. A more detailed description and validation of the skin age calculation can be found elsewhere (35,43).

#### BrainAge

Using MRI data, brain age was assessed using a validated prediction model that was previously trained on MRI scans of 32 634 UK Biobank individuals. With machine learning techniques, the discrepancy between an individual’s biological age and chronological age of the brain (“brain age gap”) was assessed (for detail, see (44)).

#### Subjective felt age

For this parameter, participants indicated how old they felt in years. In line with previous research (45,46), we calculated proportional discrepancy scores by subtracting participants’ subjective age from their chronological age and then dividing by chronological age. Positive scores indicate a younger subjective age. Following common practice (45), proportional discrepancy scores 3 standard deviations above or below the mean were considered as outliers (47).

#### Subjective life expectancy

Subjective life expectancy was assessed with a single item asking participants to estimate their age at death. Participants responded by providing a numeric value. It was adjusted for the chronological age. For further details, see (48).

#### Subjective health expectancy

Subjective health expectancy was assessed with a single item asking participants to assess up to which age they will be healthy. Participants responded by providing a numeric value. It was adjusted for chronological age. For further details, see (48).

Chronological age, subjective felt age, subjective life expectancy, subjective health expectancy, and epigenetic biomarkers were available at 2 time points, whereas SkinAge, BrainAge, and BioAge were only assessed once (at baseline).

### Statistical Procedures

In the first step, we examined the cross-sectional correlation between biomarkers of aging. In a second step, we examined change–change associations of all age indices for which longitudinal data were available. Change was calculated by subtracting the baseline assessment score from the follow-up assessment score. In a follow-up analysis, we examined the predictive validity of biomarkers for selected outcomes in smaller subsamples to determine whether they effectively predicted aging-related outcomes, specifically grip strength and overall mortality. This analysis aimed to assess the extent to which these biomarkers could serve as indicators of physical health and longevity. Data analysis was conducted in R (49). We defined alpha = 0.05 as the level of statistical significance.

## Results

### Sample Characteristics

Descriptive statistics for the variables under study and age at each assessment are reported in Supplementary Table 2. On average, our (European) participants were in their late 60s and early 70s, and there was a relatively equal distribution of men and women across most of the measures (see also Table 1).

**Table 1.** Results of Separate Growth Models Examining the Role of Each Biomarker for Grip Strength Trajectories

		Level		Change	
	*N*	Est.	*SE*	Est.	*SE*
Hannum’s clock (DNAmAA)	917	−0.045	0.051	−0.003	0.006
Horvath’s clock (DNAmAA)	917	−0.020	0.041	−0.007	0.005
7-CpG clock (DNAmAA)	1 395	0.001	0.022	−0.004	0.003
PhenoAge (DNAmAA)	917	−0.020	0.038	−0.011*	0.004
GrimAge (DNAmAA)	917	0.081	0.060	−0.019*	0.007
DunedinPACE	1 030	−2.301	1.539	−0.483*	0.179
DNAmTL (kb)	1 030	0.918	1.002	0.146	0.114
BioAge	1 516	0.076	0.232	−0.005	0.003
BrainAge	328	0.024	0.097	−0.011	0.011
SkinAge	1 238	0.471	0.179	0.006	0.022
Subjective age	1 264	−5.287*	1.722	0.196	0.220
Subjective life expectancy	1 388	0.030	0.034	0.004	0.004
Subjective health horizon	1 373	0.057	0.037	0.001	0.005

*Notes*: Grip strength modeled over time in study. Models include age and sex as additional predictors. Unstandardized estimates and standard errors presented.

* *p* < .05.

#### Associations between multidomain indicators of aging

**Figure 1.**
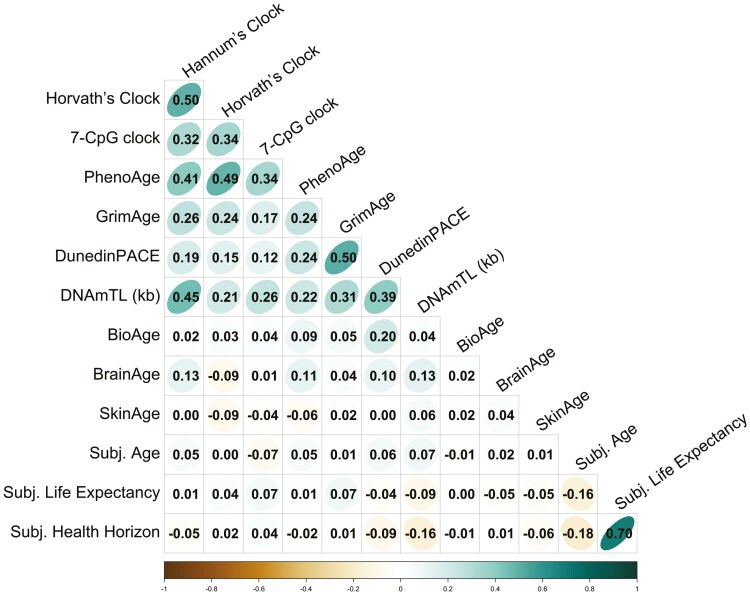
Intercorrelations among the alternative age markers at baseline assessment. The shape and fill color of the circles indicate the strength of the correlations, with more eccentric ellipses and darker circles representing higher correlations. It can be obtained that within a given domain, indicators of aging are moderately interrelated both cross-sectionally and over time. In contrast, across domains associations are rare and, if these exist, of modest size. Age indicators were scaled with regard to chronological age (exc. DunedinPACE & DNAmTL).

**Figure 2.**
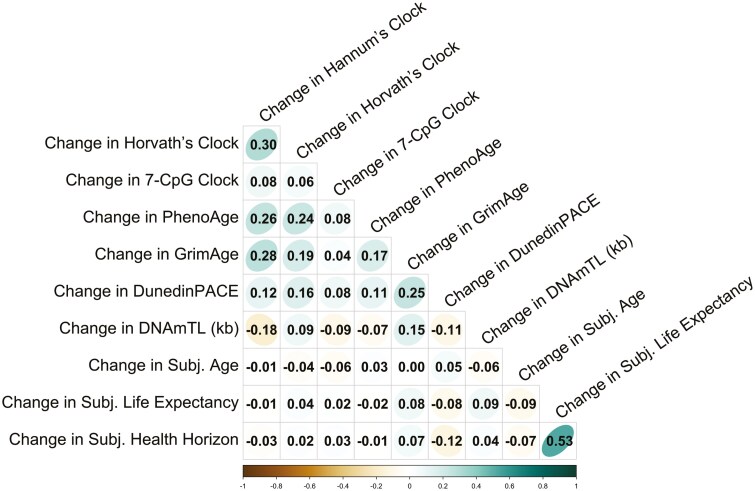
Intercorrelations among long-term longitudinal changes in the alternative age markers between baseline and follow-up assessments. The shape and fill color of the circles indicate the strength of the correlations, with more eccentric ellipses and darker circles representing higher correlations. Age indicators were scaled with regard to chronological age (exc. DunedinPACE & DNAmTL).

Intercorrelations among the 14 alternative indicators of aging are presented in Figure 1. Our analyses indicate 2 sets of findings. First, within a given domain (eg, epigenetic clocks), indicators of aging are moderately correlated. For example, the correlation between Hannum’s and Horvath’s clocks was *r* = .50, *p* < .05 (see the left-hand panel of Figure 2). Similarly, the correlation between the 2 self-reported measures of subjective life expectancy and subjective health horizon was *r* = .70, *p* < .05 (see the left-hand panel of Supplementary Figure 11).

**Figure 3.**
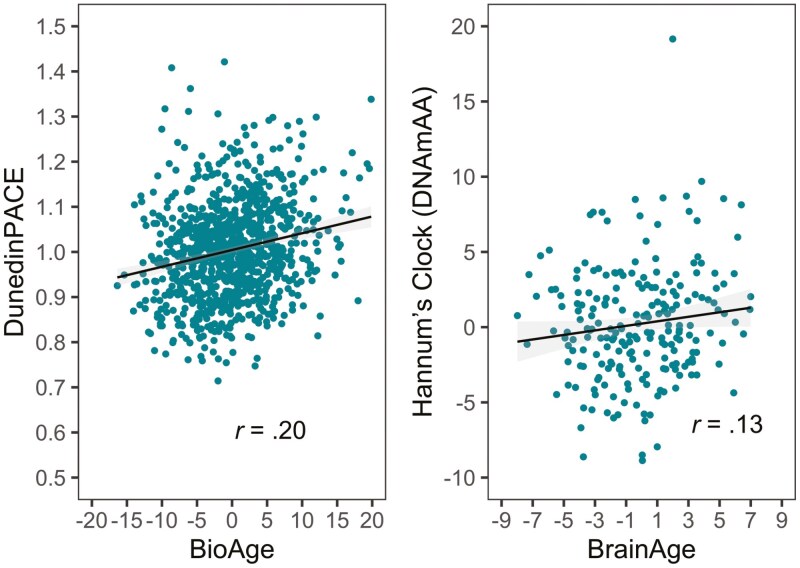
Associations between BioAge and pace of aging (DunedinPACE, left-hand panel) and between brain age and the epigenetic DNA methylation age acceleration (right-hand panel). Across domains, associations of alternative age markers are rare and, if these exist, of modest size. As notable exceptions, we found that participants with older BioAge also had a faster pace of aging (DunedinPace, left-hand panel). We also found that participants whose brain age was older than expected based on their chronological age were also older epigenetically (right-hand panel).

**Figure 4.**
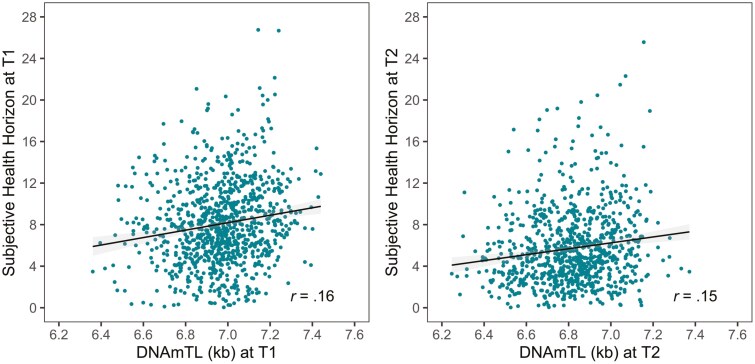
Associations between DNAm telomere length (DNAmTL) and subjective health horizon at baseline (left-hand panel) and at follow-up assessments (right-hand panel). It can be obtained that longer telomeres as assessed by DNAmTL (in kilobases, kb) are associated with wider subjective health horizons, consistently at baseline assessment (left-hand panel) and at follow-up 7 years later (right-hand panel).

Second and in contrast to this within-domain pattern, we found that across domains, associations were rare and, if they existed, of modest size. For example, participants with older BioAge also had a faster pace of aging as indicated by DunedinPACE (*r* = .20, *p* < .05, see the left-hand panel of Figure 3). Similarly, participants whose brain age was older than expected based on their chronological age were also epigenetically older, using Horvath’s clock (DNAmAA, *r* = .13, *p* < .05, see the right-hand panel of Figure 3). Finally, longer telomeres as assessed by DNAmTL (in kilobases, kb) are associated with wider subjective health horizons, consistently at baseline assessment (*r* = .16, *p* < .05, see the left-hand panel of Figure 4) and at follow-up 7 years later (*r* = .15, *p* < .05, see the right-hand panel of Figure 4). Another exception to this pattern was that we found the surrogate marker for TL, DNAmTL, to be moderately and negatively correlated with the epigenetic clock measures (DNAmAA estimated by the different epigenetic clocks and DunedinPACE; *r* between −0.21 and −0.45, *p* < .05).

Despite these sporadic nominal correlations, we did not observe any across-domain associations. For instance, skin age and subjective age were not associated with DunedinPACE and BioAge, respectively (*r*’s = .00 and −.01, respectively, *p* > .10, see Supplementary Figure 11). We basically obtained the same pattern of cross-sectional associations both at baseline assessment (see Figure 1) and follow-up assessment on average 7 years later (see Supplementary Figure 2).

#### Associations between changes in multidomain indicators of aging

Using the longitudinal assessments, we also observed a pattern of significant within-domain correlations, but only a few that were also present across domains (see Figure 2). For example, participants who experienced accelerated aging over the 7-year study period on the Horvath clock also experienced accelerated aging on the Hannum clock (DNAmAA, *r* = .30, *p* < .05; see the right-hand panel of Supplementary Figure 1). Likewise, participants whose subjective life expectancy shrank more rapidly than for others over the 7-year follow-up period also exhibited stronger declines than others in their subjective health horizon (*r* = .53, *p* < .05; see the right-hand panel of Supplementary Figure 11). In general, the prevailing pattern was similar to the cross-sectional results, that is, across-domain correlations were essentially absent for changes in alternative age markers (see also Supplementary Figure 2). We additionally repeated our analysis to also account for well-known factors that have been shown to be linked to age processes, including sex (women = 1), body mass index (BMI), smoking status (pack years), and education in years. Overall, our results remained stable (see also Supplementary Figures 13–15).

**Figure 5.**
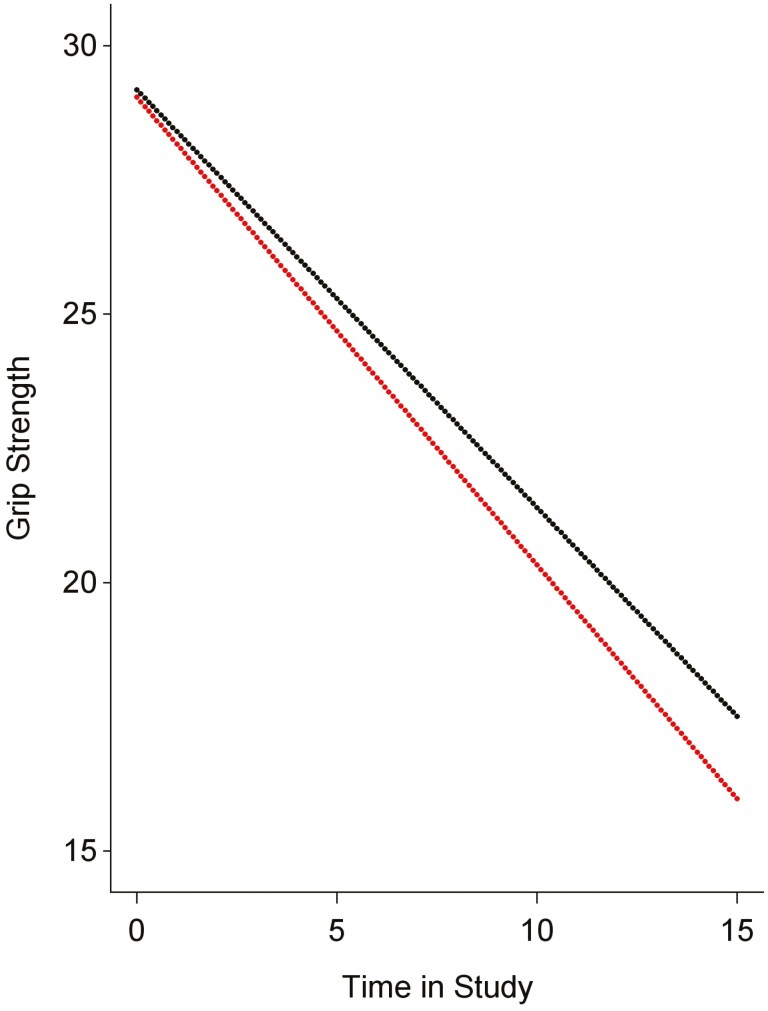
Change in grip strength over 15 years by median-split groups of participants with a faster and slower pace of aging. It can be obtained that participants who have a faster pace of aging as evinced by DunedinPACE (represented by the lower line) are experiencing steeper declines over up to 15 years in grip strength than those who have a slower pace of aging (represented by the upper line).

In follow-up analyses, we made use of recently collected information in BASE-II and demonstrated further predictive utility. These analyses revealed that, in particular, the epigenetic clocks of the second and third generations are predictive of change over time in a central index of upper-body functioning (ie, grip strength). More specifically, as can be seen in Table 1 and Figure 5, participants who have a faster pace of aging as evidenced by DunedinPACE (shown in red) are experiencing steeper declines over up to 15 years in grip strength than those who have a slower pace of aging (shown in black). Table 1 also shows that similar associations were found for PhenoAge and GrimAge, and an older subjective age was associated with poorer baseline performance on the grip strength test.

**Figure 6.**
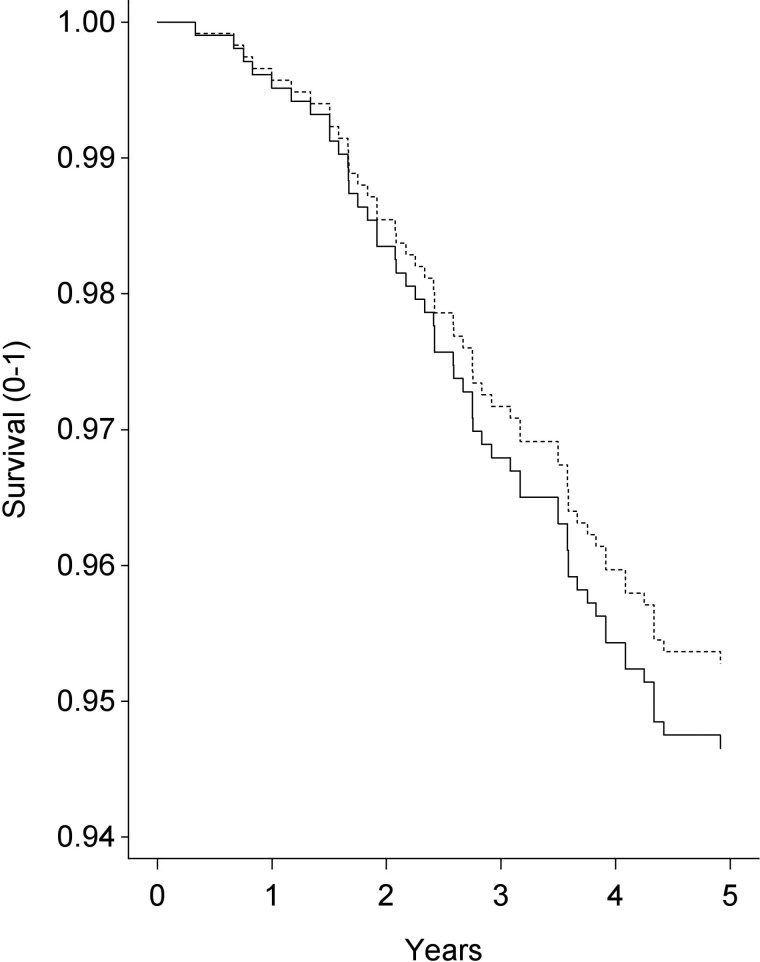
Kaplan–Meier survival curves over 5 years by median-split groups of participants with faster and slower pace of aging. It can be obtained that participants who have a faster pace of aging as evinced by DunedinPACE (shown with solid lines) are faced with lower survival when compared with participants who have a slower pace of aging (shown with dotted lines).

We also made use of the most recent mortality update information obtained in BASE-II. We interpret our results with great caution because only 5.4% (55 participants) of the analysis sample died after the follow-up examination. Yet, despite restrictions in statistical power, these first results show that our measure of DunedinPACE is associated with increased mortality hazards over and above the predictive effects of age and sex. As can be seen in Table 2 and Figure 6, participants who have a faster pace of aging estimated by DunedinPACE are faced with increased mortality hazards and thus show lower survival (solid lines) when compared with participants who have a slower pace of aging (dotted lines). On average, our participants will soon be in their early to mid-80s and thereby reach ages of high vulnerability for frailty, cognitive impairment, and mortality. At that time more thorough analyses of criterion validity in BASE-II will be possible.

**Table 2.** Results of Separate Cox Proportional Hazard Models Examining the Role of Each Biomarker for Mortality Hazards

	*N* Total	*N* Deceased	% Deceased	Hazard Ratio	95% CI
Hannum’s clock (DNAmAA)	908	50	5.5	1.027	0.797–1.323
Horvath’s clock (DNAmAA)	908	50	5.5	1.017	0.774–1.337
7-CpG clock (DNAmAA)	922	51	5.5	0.850	0.643–1.125
PhenoAge (DNAmAA)	908	50	5.5	0.978	0.739–1.293
GrimAge (DNAmAA)	908	50	5.5	1.175	0.890–1.552
DunedinPACE	1 020	55	5.4	1.333*	1.030–1.727
DNAmTL (kb)	1,020	55	5.4	0.778	0.590–1.026
BioAge	995	52	5.2	1.190	0.898–1.577
BrainAge	255	13	5.1	1.015	0.597–1.725
SkinAge	798	46	5.8	1.106	0.819–1.494
Subjective age	954	53	5.6	0.921	0.702–1.208
Subjective life expectancy	1,021	55	5.4	0.876	0.658–1.166
Subjective health horizon	1,014	54	5.3	0.953	0.712–1.275

*Notes*: Models include age and sex as additional predictors.

* *p* < .05.

## Discussion

This study investigated how different age indicators derived from blood assays, MRI brain scans, other-ratings, and self-reports converge and diverge within older adults and how these constellations change with age. Overall, we only identified moderate correlations within specific domains of aging indicators, both cross-sectionally and longitudinally. In contrast, associations across domains were infrequent and modest in size, with the exception of the epigenetic clock measures (DNAmAA and DunedinPACE) and a DNAm-based surrogate marker for TL, DNAmTL, which were moderately correlated. The pattern was similar regardless of whether cross-sectional or longitudinal data were used. Importantly, the (non)correlations found in this study are in line with correlations found in previous studies (39,50).

Given our study’s design, we can only draw inferences about sample-level associations. It is possible that a different pattern of findings emerges when one considers subgroups of people, particularly those at risk for functional decrements and impending death. We note that in follow-up analyses, we corroborated the general pattern of results among those who suffered from 2 and more chronic physical diseases at follow-up. At the same time, the BASE-II sample is still relatively healthy, so results may not generalize to participants with poor and debilitating health conditions. One way to interpret our findings is thus that the diverse and loosely interconnected pattern of aging indicators may be a defining characteristic of normal if not healthy aging. It might only be when these normal aging processes are more and more shaped by secondary aging, involving pathological changes, and tertiary aging, culminating in mortality, that the heterogeneous profile transitions into a more uniform and homogeneous pattern characterized by generalized broad-based declines (46,47). It stands to reason that refined measures will help monitor how such transitions evolve.

Research on the associations between psychological and biological indicators of aging is extremely scarce. Even though a number of studies have highlighted the importance of physiological health for subjective age and vice versa (51,52), we are unaware of any study examining links between subjective and biological/epigenetic age indicators. Interestingly, stronger epigenetic age acceleration was associated with a narrower subjective health horizon, consistently at baseline and follow-up 7 years later. It has been argued that an individual’s sense of aging can be shaped by aggregate cellular stress (53). Individuals who have undergone challenging life experiences may perceive themselves as prematurely aged due to the cumulative impact of subclinical deterioration and allostatic load. Research has demonstrated that the allostatic load, which reflects the wear and tear on the body resulting from prolonged exposure to stressors, is linked to epigenetic age measures (54). Conversely, psychological stress, a significant contributor to allostatic load, has been found to be associated with an older subjective age (55). Also, it was surprising to see that skin age was not strongly linked to subjective age even though one might assume that visible signs of aging would be internalized and affect how old someone feels (56,57). The validity of the subjective age measure used in this article has been shown in a number of studies (52), and so has the validity of the skin age measure (35,43). We note that skin age was estimated based on the number and distribution of lentigines at the back of the hands; possibly, the occurrence of facial lentigines may be more strongly associated with subjective age ratings. Not surprisingly, we found moderate negative correlations between the epigenetic clock measures (DNAmAA) and the surrogate marker for TL, DNAmTL, which were in the similar range previously reported by Lu and colleagues (38).

We also note that there is currently no gold standard on how to measure biological age. Therefore, we analyzed a number of different aging indicators, many of which represent the current state-of-the-art in quantifying the pace of aging. At the same time, we acknowledge that these measures do not represent an exhaustive list of biological changes that evolve with aging and also come with a number of technical and methodological limitations (26,58,59). For example, the variables analyzed often indicate processes that evolve on different (time) scales. To illustrate, some markers like TL (DNAmTL) track the actual progress of age, whereas others are calculated by taking the difference between the measured (=“biological”) age and the chronological age (eg, DNAmAA). This means that some measures are tied to chronological age, whereas others are not. We also note that the age markers compared here are different in the underlying conceptual rationale and were in part optimized using different criteria. For instance, some biomarkers were developed and optimized to predict future mortality and others were developed and optimized to estimate concurrent chronological age or biological age. Such conceptual and methodological differences may reduce the strength of associations, yet one would presumably still expect to find sizeable interrelations.

To our knowledge, no study so far has examined longitudinal associations between changes in multidomain indicators of aging. Our findings indicate that correlations between changes in age indicators were very small or absent. For example, changes in epigenetic age measures were not related in statistically significant ways to changes in any other indicators of aging. Such absence of across-domain associations was unexpected because it appears plausible that people who show an age acceleration in one domain of functioning (as proxied by one set of alternative age markers) should show similar trends in other domains of functioning (as proxied by another set of alternative age markers). These findings raise intriguing questions about the underlying mechanisms and methodological considerations that may contribute to the lack of association.

One explanation could be the inherent complexity of aging processes themselves. Aging is a multifaceted phenomenon influenced by a variety of different biological, environmental, experiential, and behavioral factors, each contributing to different aspects of people’s aging trajectories. It is plausible that changes in epigenetic age measured from leukocyte DNA may capture distinct biological processes that are not fully reflected in other measures of aging, resulting in divergent trajectories over time. Another methodological consideration is the measurement sensitivity and specificity of the different aging indicators. Each indicator represents a different facet of aging, and their sensitivity to capture changes may vary. It’s worth noting that the cross-sectional correlation between GrimAge and DunedinPACE is nearly double that of the longitudinal correlation (*r* = .50 vs *r* = .25). This finding is presumably due to the fact these clocks capture distinct aspects of aging. GrimAge reflects how much older or younger an individual epigenetically is compared to their chronological age, serving as a level index. DunedinPACE, on the other hand, is a change index that reflects the rate at which a person is aging. While these measures may correlate in a cross-sectional analysis, their longitudinal changes may not align, as a quicker change in one could correspond to stability in the other. It is, thus, possible that the specific metrics used might not fully align with the changes observed in other indicators, leading to the lack of association between them. Furthermore, the time frame and duration of the study may have influenced the observed associations. Aging encompasses dynamic processes that evolve over extended periods of time, and changes in aging indicators may not manifest in synchronous ways. Longitudinal studies with longer follow-up periods and more frequent assessments may provide a more comprehensive understanding of associations between changes in different aging indicators. For example, it remains unclear whether changes in aging indicators follow a linear trajectory or exhibit a nonlinear pattern. Trajectories might not be perfectly aligned and thus the timing of examinations will impact whether or not associations are found. It is also possible that the strength of correlations changes across the lifespan. Because aging involves cumulative processes, the impact of certain biomarkers may intensify and magnify in older ages and longitudinal associations between these biomarkers may be more likely to be observed toward the end of life and less so in midlife or earlier phases of old age.

### Study Limitations and Outlook

In closing, we note the following limitations of our study design, measures, and samples. First, not all of the clocks determined at baseline had been assessed at the same point in time. For example, on average there were more than 2 years in between assessments of the epigenetic clocks and brain age (*M* = 2.28, *SD* = 1.19). We note, however, that we observed similar patterns of noncorrelations for clocks that had been assessed within just a few days (ie, participants filled out the questionnaire data in between the 2 medical sessions during which blood samples were taken). It, thus, appears unlikely that the nonsimultaneous assessment of some of the measures constitutes a major contributing factor to the noncorrelations observed here. We also note that our study design does not allow us to examine the trajectories of markers of aging and how they are associated with one another. Given that we only had 2 time points available over the course of on average 7 years, we cannot draw further conclusions about the speed and shape of change. For example, it is possible that certain health events during the 7-year period have led to an accelerated decline in some individuals, whereas others might have experienced a steady change throughout the 7 years. Future research needs to further examine the shape of aging trajectories.

We also note that with the availability of only 2 time points, measurement error could affect our results, introducing inaccuracies that might skew the observed relationships between variables. This limitation underscores the necessity for additional research to comprehensively address and mitigate the impact of measurement error, which can impact the validity and reliability of our findings. Evidence from recent studies has shown considerable variability in the test–retest reliability of DNAm clock age-residuals across repeated assays of the same DNA samples (26,60,61). Specifically, the Horvath, Hannum, and PhenoAge clocks have demonstrated moderate-to-poor reliability, whereas GrimAge and DunedinPACE clocks exhibited better reliability (26). Similarly, extensive literature has discussed the reliability of TL and also subjective age measures, indicating that, despite some variability, results are generally satisfactory (62,63). Future studies should move toward collecting and analyzing multiple waves of within-person change data preferably from multiple indicators per construct and thereby allow employing robust statistical techniques that take into account and correct for unreliability of measurement.

Changes in some biomarkers may serve as antecedents or predictors of changes in other biomarkers. For instance, epigenetic biomarkers may precede or influence the development of clinical or psychological biomarkers of aging (eg, older epigenetic and BioAge might precede older skin age). It will be interesting to assess such time-ordered associations in future work.

Similarly, consider the potential scenario where epigenetic biomarkers of aging, such as specific DNAm patterns, precede and influence the development of clinical or psychological biomarkers of aging. For instance, alterations in DNAm patterns associated with accelerated aging may be antecedents to cognitive decline or the manifestation of age-related diseases. While such associations were reported in the literature, no statistically significant associations were found between epigenetic clocks and a number of functional, geriatric, and cognitive assessments or frailty in BASE-II before (39,42). In this case, understanding the antecedent biomarkers can provide valuable insights into the underlying mechanisms of aging-related conditions and potentially guide targeted interventions for prevention or early intervention. Hence, identifying the specific biomarkers that have a stronger association with specific clinical outcomes becomes crucial for effective clinical management and personalized interventions.

We note that this example represents only a single potential scenario, and the relationships and temporal dynamics between different biomarkers of aging are likely complex and context-dependent. For example, we found a negative correlation between baseline and subsequent change in several of our alternative aging measures. As can be seen in the Supplementary Material (see Supplementary Figure 16), subjective age and subsequent change in subjective age were correlated with *r* = −.44. Such a pattern presumably results from multiple contributing factors, including regression to the mean, more room to change (ie, to lose) for those who were initially in very good shape, or compensatory biological responses through homeostatic regulation. Such a pattern of findings provides yet another impetus for our current endeavors to collect a third wave of data that will eventually allow us to move toward using latent growth curve modeling that accommodate some of the problems by estimating change in latent space. Importantly, the interplay among biomarkers and their time-ordered or even causal pathways can vary based on the specific aging process or disease under investigation. Thus, generalizing results from one scenario to other biomarkers or aging processes should be done with caution. To gain a comprehensive understanding of the intricate associations between biomarkers of aging, longitudinal studies with large and diverse populations are needed that elucidate the complex interdependencies and causal pathways among various biomarkers. For example, variance observed in BrainAge has been shown to have ontogenetically early sources, indicating that it does not solely reflect differences that develop during adulthood (64–66). Integrating multiple levels of data, including epigenetic, proteomic, and clinical measures, will likely provide a more comprehensive picture of the aging process (42,67).

Also, the aim of this article was to describe interrelations among multidomain age indicators. To better understand aging processes, one next step will be to examine their unique, conjoint, and interactive effects in predicting clinical outcomes. For example, studies have highlighted the predictive validity of alternative age indicators for substance use behaviors, atherosclerosis, cancer, and overall mortality (58,68,69). It remains a largely open question which of a multitude of biomarkers are most predictive of which outcome when examined together. We also acknowledge that while more comprehensive than most other studies our selection of biomarkers was limited by data availability. For example, BioAge, skin age, or BrainAge were not available at follow-up not allowing us to examine change–change associations. Further research is needed to close this gap. Importantly, the field of biomarkers of aging is still evolving, and ongoing research continues to uncover new biomarkers and further elucidate their role in aging processes.

Finally, we used data from the sample of the BASE-II, which is restricted on several dimensions. First, the dataset was recruited within a geographically restricted area encompassing Berlin and its surroundings. Specifically, our sample lacked racial diversity, making it challenging to quantify the generalizability of our results. As a consequence, we can draw no inferences from other, more diverse populations. This remains an important limitation as previous work has suggested that there are disparities in aging patterns among different racial and ethnic groups. For instance, research indicates that non-Hispanic Blacks and Hispanics may exhibit accelerated aging, whereas non-Hispanic Whites (ie, the ethnic group studied here) may experience decelerated aging (59). At the same time, it is important to acknowledge that these racial and ethnic differences are intricately linked to other factors, such as educational attainment. Thus, it is important for future research to replicate and validate our findings in more geographically and racially/ethnically diverse samples.

## Conclusion

Here we tested whether and how a relatively broad array of 14 different aging clocks derived from a diverse set of domains correlate in older adults in both cross-sectional and longitudinal assessments. Our results suggest that within a given domain (eg, epigenetic clocks based on DNAm), indicators of aging are moderately correlated both cross-sectionally and longitudinally. In contrast, across domains associations were mostly absent and, if they existed, of only modest size. For example, skin age and subjective age did not show any correlations with faster epigenetic age acceleration and BioAge, respectively. Our results indicate that there are multiple clocks that time us and that it is important to consider multiple indicators of age and aging when trying to understand the heterogeneity of aging.

## Supplementary Material

glae244_suppl_Supplementary_Material

## Data Availability

The data presented in this study are available from the BASE-II office, please see https://www.base2.mpg.de/7549/data-documentation for details. We are not in a position to make data publicly available because these contain information that could compromise research participants’ privacy and consent. Our statistical analysis code has been made publicly available in the Supplementary Material (Section Statistical Analysis Code).
