# Supplementary material for: There Are Multiple Clocks That Time Us: Cross-Sectional and Longitudinal Associations Among 14 Alternative Indicators of Age and Aging
Source: J Gerontol A Biol Sci Med Sci. 2024 Oct 9;80(6):glae244. doi: 10.1093/gerona/glae244 (PMC12104808; doi:10.1093/gerona/glae244)

There are multiple clocks that time us: Cross-sectional and longitudinal associations among 14 alternative indicators of age and aging

Johanna Drewelies, Ph.D.[1], Jan Homann, M.Sc.[2], Valentin Max Vetter, M.D.[3], Sandra Düzel, Ph.D.[3], Simone Kühn, Ph.D.[1,5], Laura Deecke, M.Sc.[2], Elisabeth Steinhagen–Thiessen, M.D.[3], Philippe Jawinski, Ph.D.[4], Sebastian Markett, Ph.D.[4], Ulman Lindenberger, Ph.D.[1,5], Christina M. Lill, M.D.[2,6], Lars Bertram, M.D.[7], Ilja Demuth, Ph.D.[3*], & Denis Gerstorf, Ph.D.[4,8*]

[1]Max Planck Institute for Human Development Berlin
[2]Institute of Epidemiology and Social Medicine, University of Münster
[3]Charite – Universitätsmedizin Berlin
[4]Humboldt Universität zu Berlin
[5]Max Planck UCL Centre for Computational Psychiatry and Ageing Research
[6]Ageing Epidemiology Unit, School of Public Health, Imperial College London
[7]University of Lübeck
[8]German Institute for Economic Research, DIW Berlin

*joined last authors

Author Note

Johanna Drewelies https://orcid.org/0000-0002-3774-2169; Jan Homann https://orcid.org/0000-0003-2791-7065; Valentin Max Vetter https://orcid.org/0000-0001-5003-7766; Sandra Duezel https://orcid.org/0000-0001-7179-2664; Simone Kühn https://orcid.org/0000-0001-6823-7969; Laura Deecke https://orcid.org/0009-0002-9268-2469; Elisabeth Steinhagen–Thiessen https://orcid.org/0000-0003-3056-3317; Philippe Jawinski https://orcid.org/0000-0002-2994-3075; Sebastian Markett https://orcid.org/0000-0002-0841-3163; Ulman Lindenberger https://orcid.org/0000-0001-8428-6453; Christina Lill https://orcid.org/0000-0002-2805-1307; Lars Bertram https://orcid.org/0000-0002-0108-124X; Ilja Demuth https://orcid.org/0000-0002-4340-2523; Denis Gerstorf https://orcid.org/0000-0002-2133-9498

Online Supplementary Material

Table OSM.1. Selected Examples of Biomarker of Aging Validation Studies*

Biomarker	Validation outcome	Reference
Hannum's Clock (DNAmAA)	frailty	Gale et al., 2018
	morbidity	Lu et al., 2019
	mortality	Lu et al., 2019
Horvath's Clock (DNAmAA)	frailty	Breitling et al. 2016
	cancer cells, somatic mutations	Horvath, 2013
	vitamin D status & supplementation	**Vetter et al., 2020, 2022**
	morbidity	Lu et al., 2019
7-CpG clock (DNAmAA)	vitamin D status & supplementation	**Vetter et al., 2020, 2022**
	cardiovascular health	**Lemke et al., 2022**
	risk for developing additional diabetes complications	**Vetter et al., 2023**
PhenoAge (DNAmAA)	morbidity	Lu et al., 2019
	frailty	Verschoor et al., 2021
	metabolic syndrome	**Demuth et al., 2022**
	subjective perceptions of aging	Belsky, 2022
	cognitive capacity	Belsky, 2022
	mortality	Lu et al., 2019
GrimAge (DNAmAA)	physical health (grip strength, lung function), mental health; morbidity	Lu et al., 2019
	frailty	McCrory et al., 2021, Verschoor et al., 2021
	metabolic syndrome	**Demuth et al., 2022**
	subjective perceptions of aging	Belsky, 2022
	cognitive capacity	Belsky, 2022
	mortality	Lu et al., 2019
DunedinPACE	brain structure	Whitman, 2024
	mortality	Belsky, 2022; Faul et al., 2023
	cognitive decline	Reed et al., 2022; Sugden et al., 2022
	morbitity	Belsky, 2022; Faul et al., 2023
BioAge	mortality	Levine et al., 2013; **Drewelies et al., 2022**

	morbidity	**Drewelies et al., 2022**
BrainAge	cognitive health	**Jawinski et al., 2022**
		Franke & Gaser, 2019
SkinAge	nonfacial solar lentigines	**Deecke et al., 2024**
Subjective Age	mortality	Westerhof et al., 2023; Kotter-Grühn et al., 2009
	physical health, cognitive health	Debreczeni & Bailey, 2021; **Notthoff et al., 2018**
Subjective Life Expectancy	MRI-based brain regions	**Duezel et al., 2018**
	physical health, cognitive health	**Duezel et al., 2016**
Subjective Health Horizon	MRI-based brain regions	**Duezel et al., 2018**
	physical health, cognitive health	**Duezel et al., 2016**

Note. References noted in bold are based on using data from the Berlin Aging Studies.

*We recognize that providing a comprehensive quantitative meta-analysis for each biomarker goes beyond the scope of our empirical report. We acknowledge yet that effect sizes for such associations certainly vary between biomarkers. For example, Horvath clock residuals are associated with mortality risk in some studies, but overall, the effect-size is about 1/10th of that of GrimAge, see[34]).

.

Figure OSM.1. *Associations between epigenetic clock indicators.*

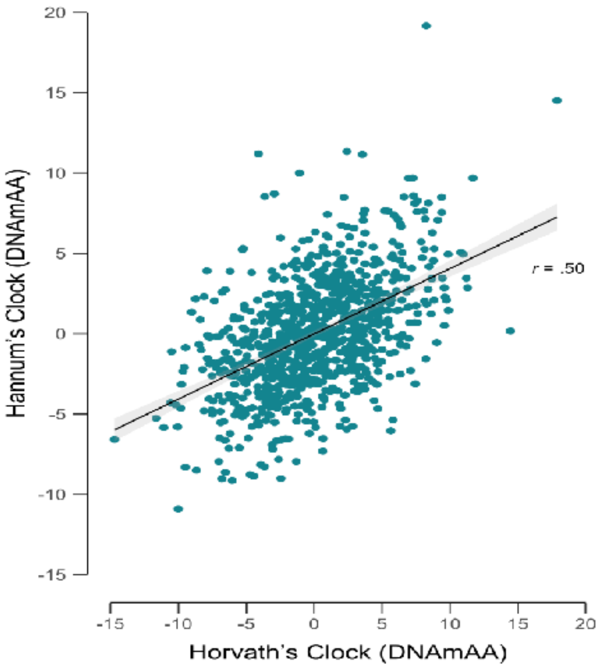

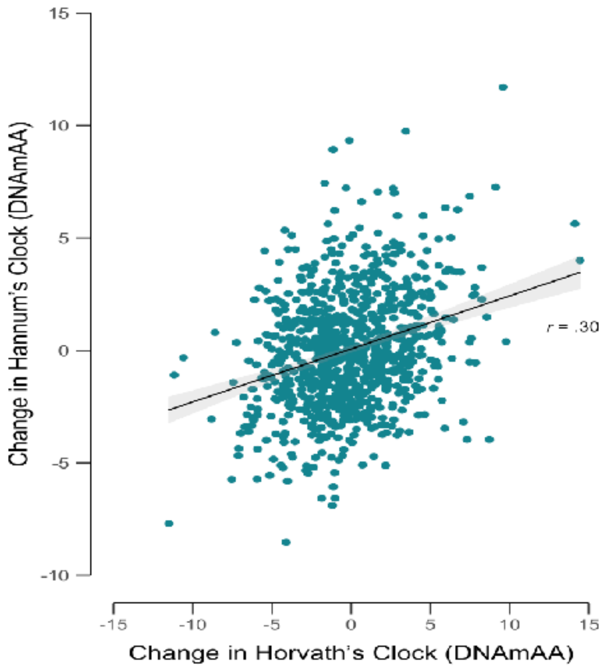

Note. It can be obtained that within a given domain (here, DNA methylation age acceleration, DNAmAA, determined from epigenetic clocks), indicators of aging are moderately interrelated both cross–sectionally and over time.

Table OSM.2. *Descriptive Statistics for the Variables Under Study and Age at Assessment*

	Mean	SE	N	*Mean* Age	*SD* Age	*% women*
Time difference between T1 & T2	7.348	0.0444	1,517	-	-	-
Age	68.26	3.470	1,517	-	-	-
Women	0.508	0.011	1,517	68.27	3.47	-
Education (in years)	14.272	0.706	1,517	70.62	3.86	50%
BMI	26.816	0.104	1,517	68.27	3.47	50%
Smoking (pack years)	10.384	0.439	1,517	68.27	3.47	50%
Hannum’s Clock (DNAmAA)	0.006	0.115	917	68.26	3.48	50%
Horvath’s Clock (DNAmAA)	0.035	0.140	917	68.26	3.48	50%
Horvath’s Clock (DNAmAA, buccal)	0.000	6.746	844	75.76	3.76	53%
7-CpG clock (DNAmAA)	0.018	0.185	1,395	68.76	3.71	49%
PhenoAge (DNAmAA)	0.035	0.152	917	68.26	3.48	50%
GrimAge (DNAmAA)	0.011	0.102	917	68.26	3.48	50%
DunedinPACE	1.006	0.003	1,030	68.27	3.47	51%
DNAmTL (kb)	6.969	0.006	1,030	68.27	3.47	51%
BioAge	0.000	0.157	1,517	68.70	3.63	52%
BrainAge	–0.010	0.176	328	70.54	3.86	39%
SkinAge	1.724	0.026	1,240	68.89	3.73	54%
Subjective Age	–0.130	0.002	1,265	70.52	3.81	53%
Subjective Life Expectancy	10.563	0.119	1,389	70.62	3.86	51%
Subjective Health Horizon	7.880	0.111	1,374	70.62	3.85	51%

*Note: GrimAge (DNAmAA), Hannum’s Clock (DNAmAA), Horvath’s Clock (DNAmAA), Horvath’s Clock (DNAmAA, buccal), 7-CpG clock (DNAmAA), 7-CpG clock (DNAmAA), PhenoAge (DNAmAA) and BioAge were age residualized.

Figure OSM.2. *Intercorrelations among the alternative age markers at follow-up assessment, if available.*

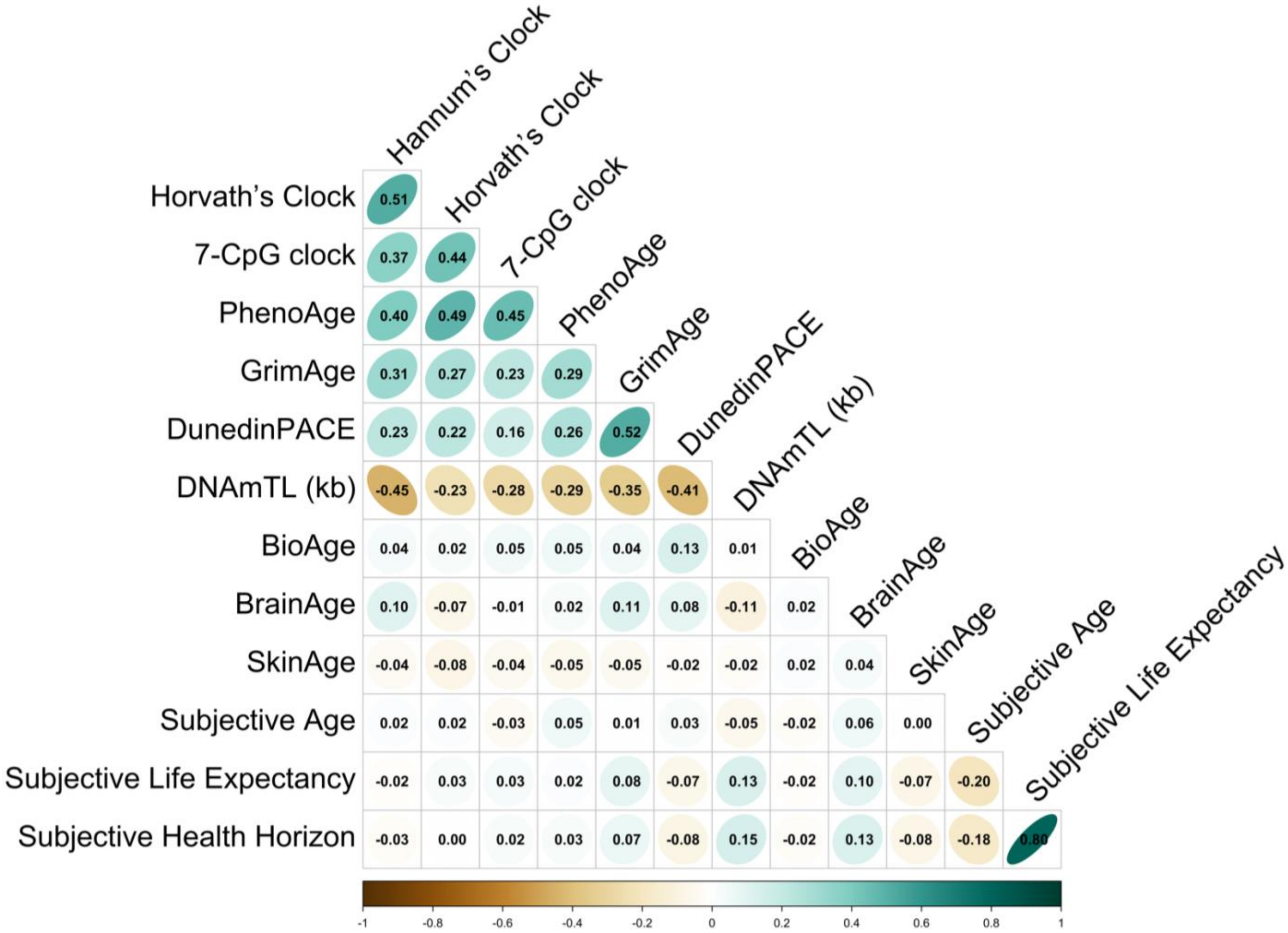

Note. Data for BioAge, BrainAge, and SkinAge taken from baseline assessment. The shape and fill color of the circles indicate the strength of the correlations, with more eccentric ellipses and darker circles representing higher correlations. Positive correlations are represented by green circles and negative correlations are represented by brown circles. Age indicators were scaled with regard to chronological age (exc. DunedinPACE & DNAmTL).

Figure OSM.3. *Intercorrelations among the alternative age markers at follow-up assessment (using Horvath's Clock (DNAmAA, buccal swap DNA).*

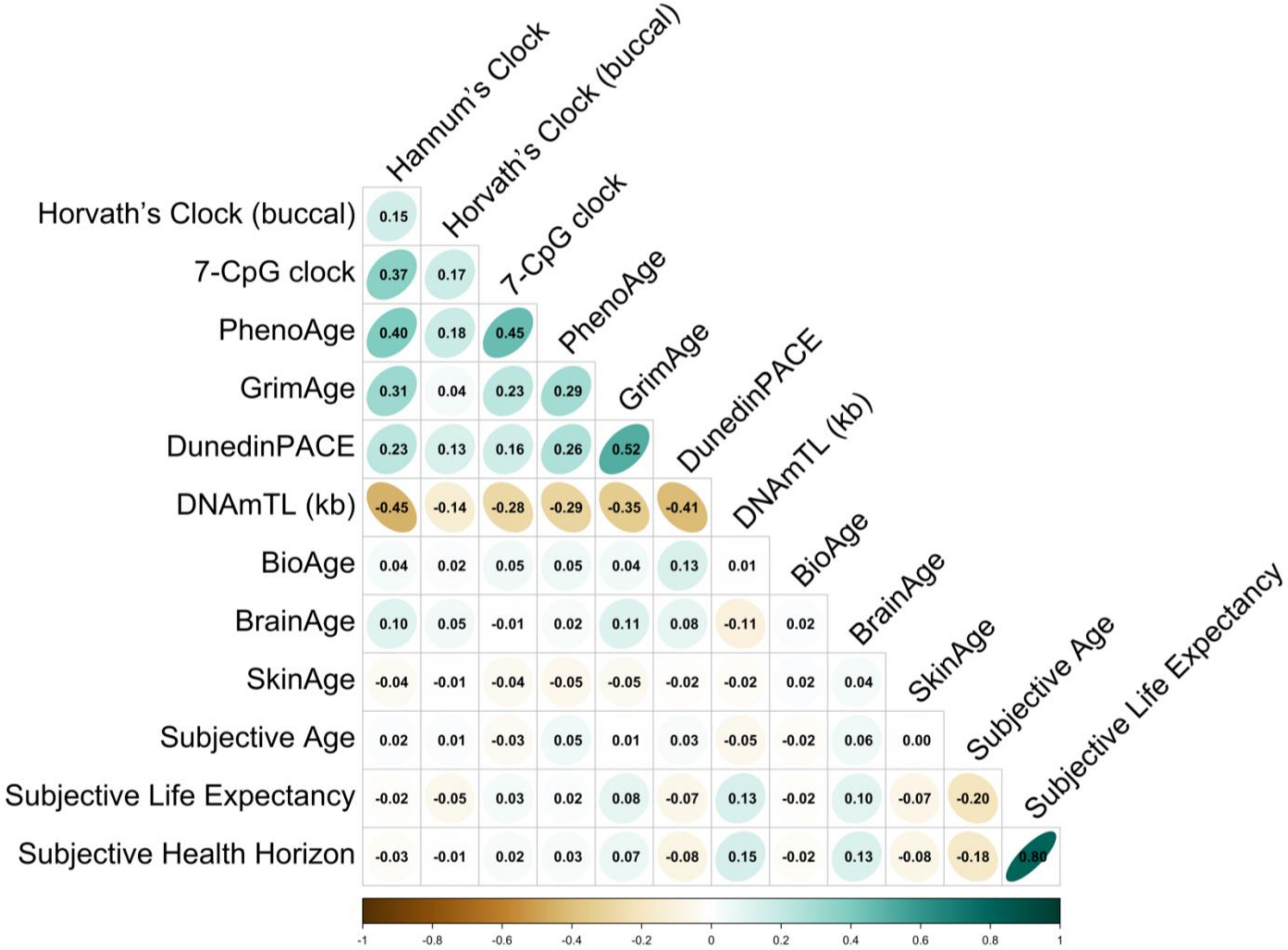

Note. Data for BioAge, BrainAge, and SkinAge taken from baseline assessment. The shape and fill color of the circles indicate the strength of the correlations, with more eccentric ellipses and darker circles representing higher correlations. Positive correlations are

represented by green circles and negative correlations are represented by brown circles. Age indicators were scaled with regard to chronological age (exc. DunedinPACE & DNAmTL).

Figure OSM.4. *Intercorrelations between the alternative age markers at baseline assessment and long-term longitudinal changes.*

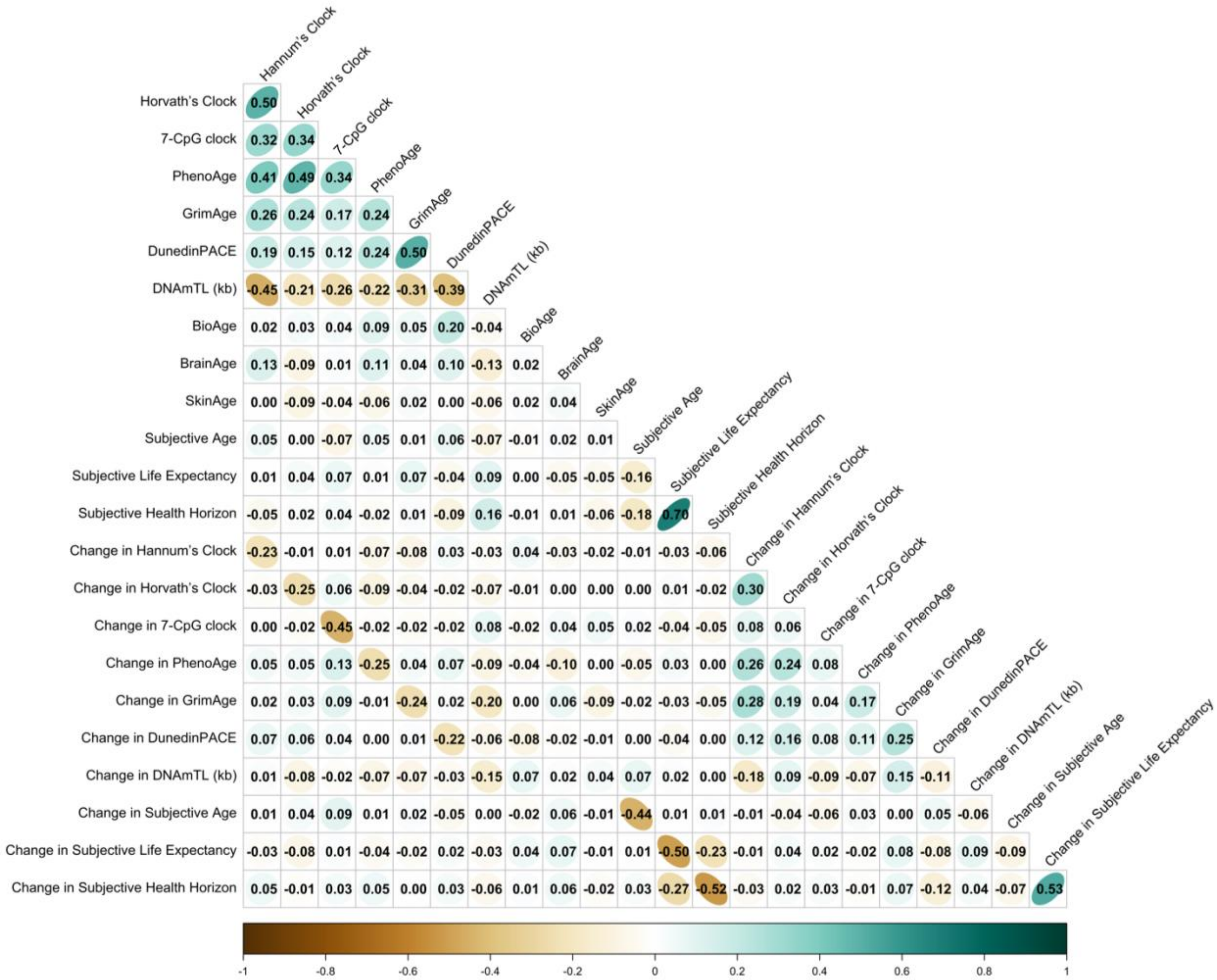

Note. Intercorrelations that were not statistically significant at $p < .05$ were omitted for clarity. The shape and fill color of the circles indicate the strength of the correlations, with more eccentric ellipses and darker circles representing higher correlations. Positive correlations are represented by green circles and negative correlations are represented by brown circles. Age indicators were scaled with regard to chronological age (exc. DunedinPACE & DNAmTL).

Figure OSM.5. *Intercorrelations among the alternative age markers at follow-up assessment using complete cases.*

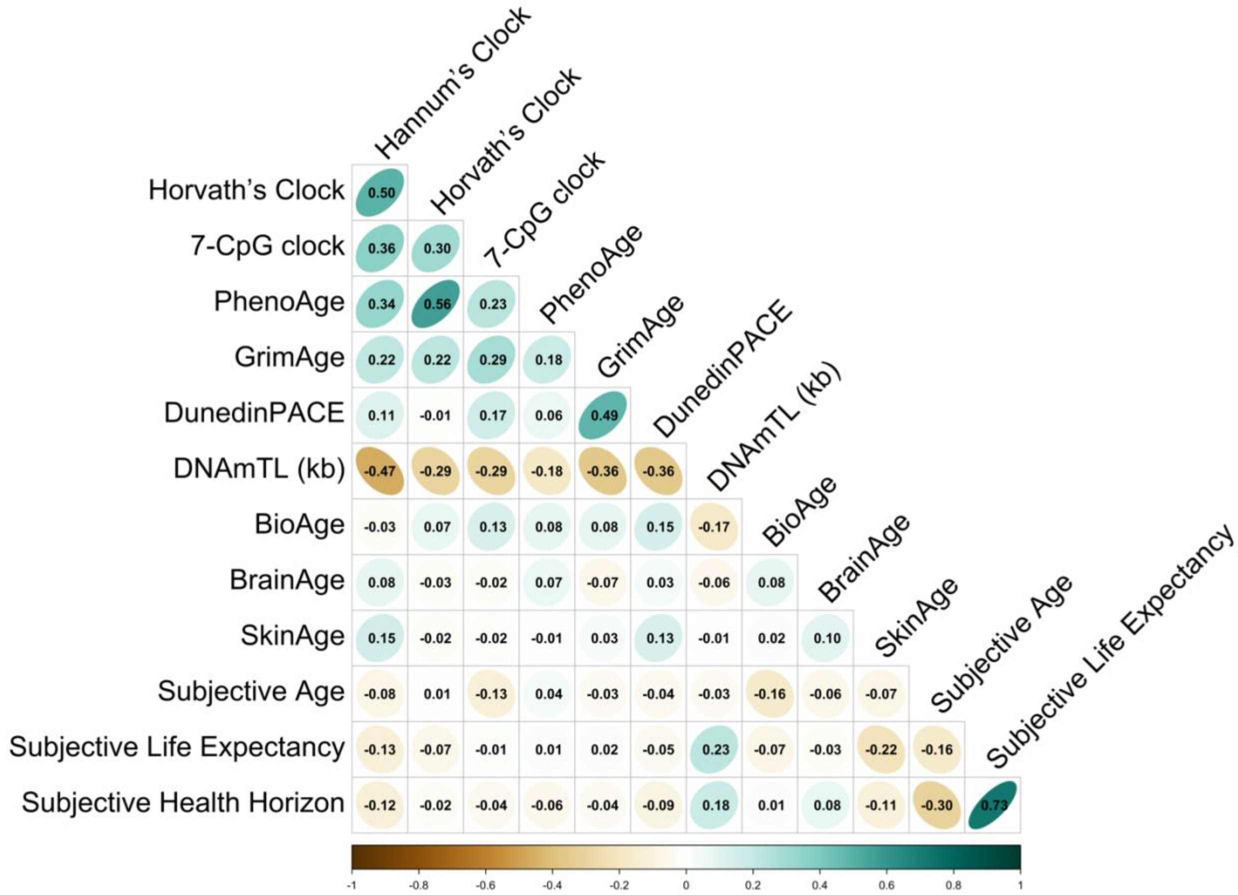

Note. We want to point out that the correlation between epigenetic clocks with the exception of the DunedinPACE methylation age was already published as part of [24]. The shape and fill color of the circles indicate the strength of the correlations, with more eccentric ellipses and darker circles representing higher correlations. Positive correlations are represented by green circles and negative correlations are represented by brown circles. Age indicators were scaled with regard to chronological age (exc. DunedinPACE & DNAmTL).

Figure OSM.6. *Intercorrelations among the alternative age markers at follow-up assessment using complete cases.*

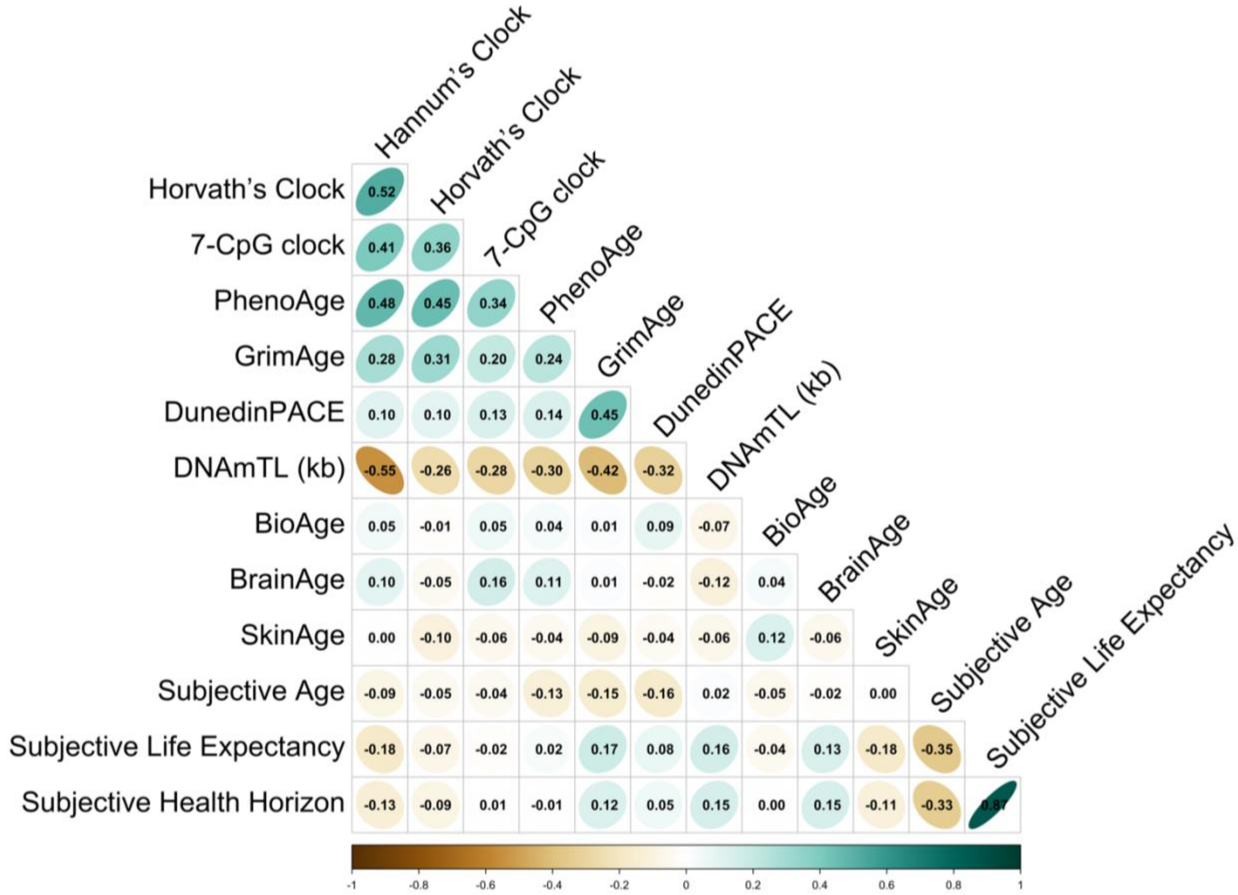

Note. Data for BioAge, BrainAge, and SkinAge taken from baseline assessment. The shape and fill color of the circles indicate the strength of the correlations, with more eccentric ellipses and darker circles representing higher correlations. Positive correlations are represented by green circles and negative correlations are represented by brown circles. Age indicators were scaled with regard to chronological age (exc. DunedinPACE & DNAmTL).

Figure OSM.7. *Intercorrelations among the alternative age markers at baseline assessment for women.*

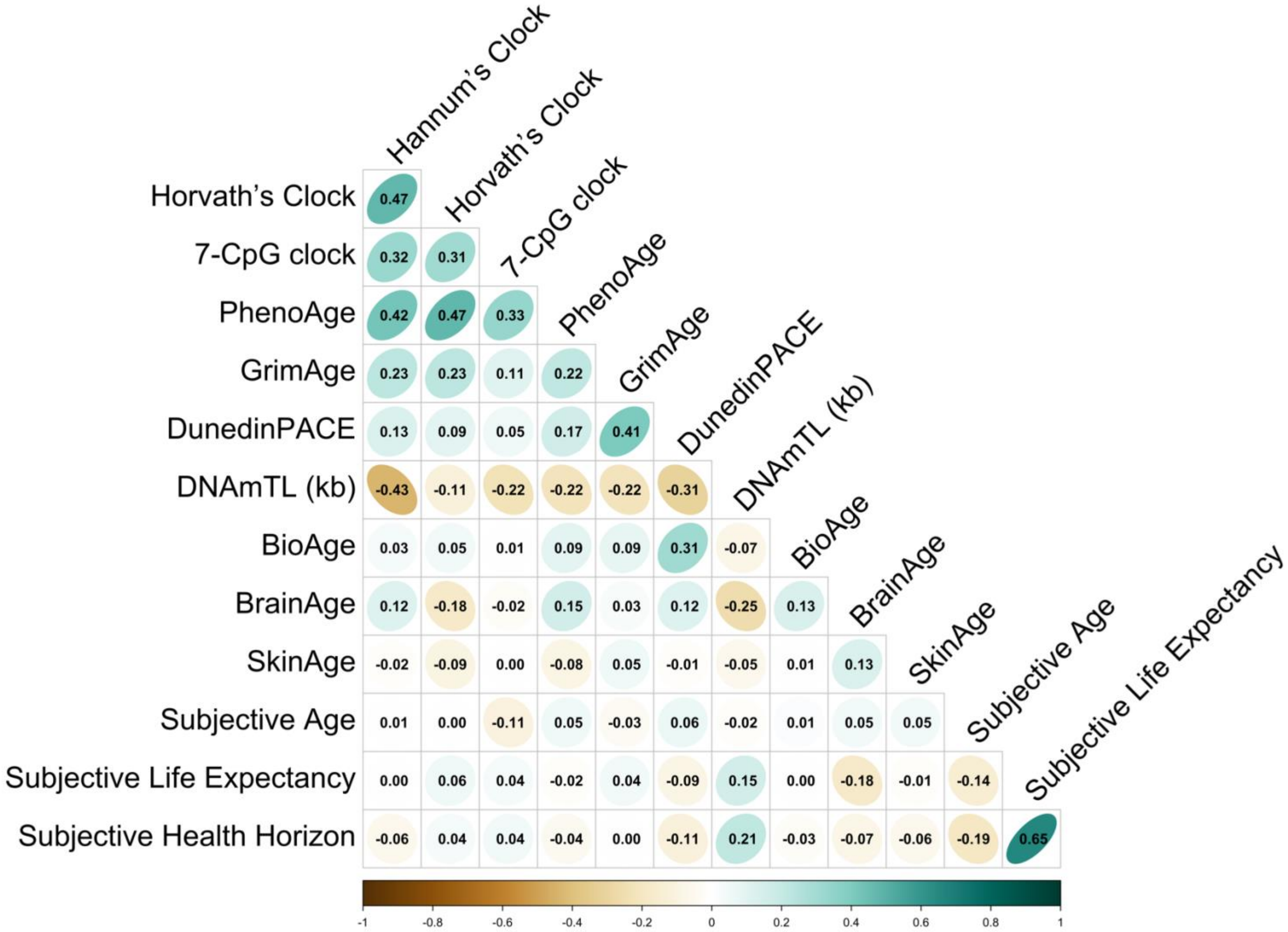

Note. The shape and fill color of the circles indicate the strength of the correlations, with more eccentric ellipses and darker circles representing higher correlations. Positive correlations are represented by green circles and negative correlations are represented by brown circles. Age indicators were scaled with regard to chronological age (exc. DunedinPACE & DNAmTL).

Figure OSM.8. *Intercorrelations among the alternative age markers at baseline assessment for men.*

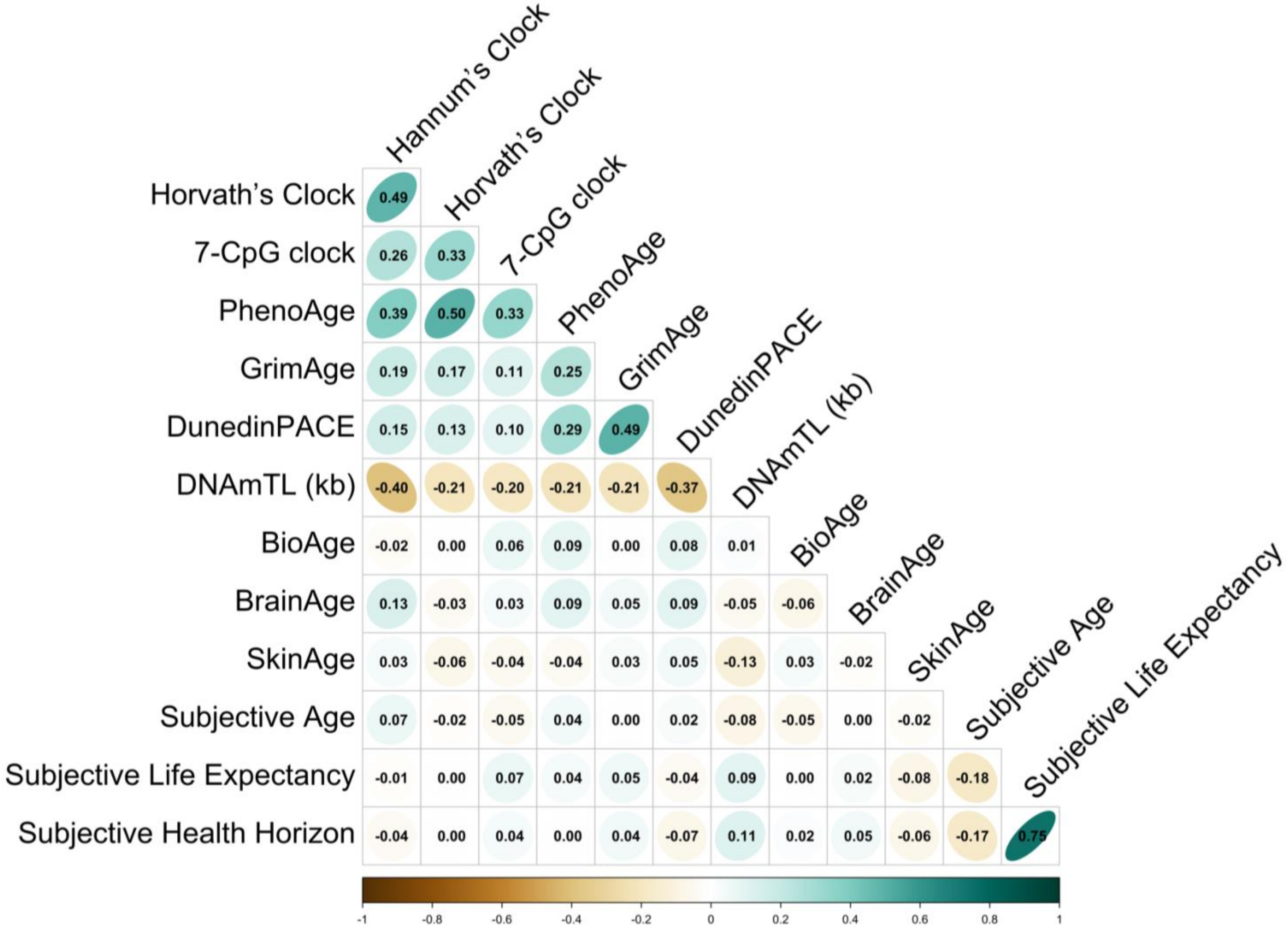

Note. The shape and fill color of the circles indicate the strength of the correlations, with more eccentric ellipses and darker circles representing higher correlations. Positive correlations are represented by green circles and negative correlations are represented by brown circles. Age indicators were scaled with regard to chronological age (exc. DunedinPACE & DNAmTL).

Figure OSM.9. *Intercorrelations among the alternative age markers at follow-up assessment for those who suffered from two and more chronic physical diseases at follow-up (n = 376).*

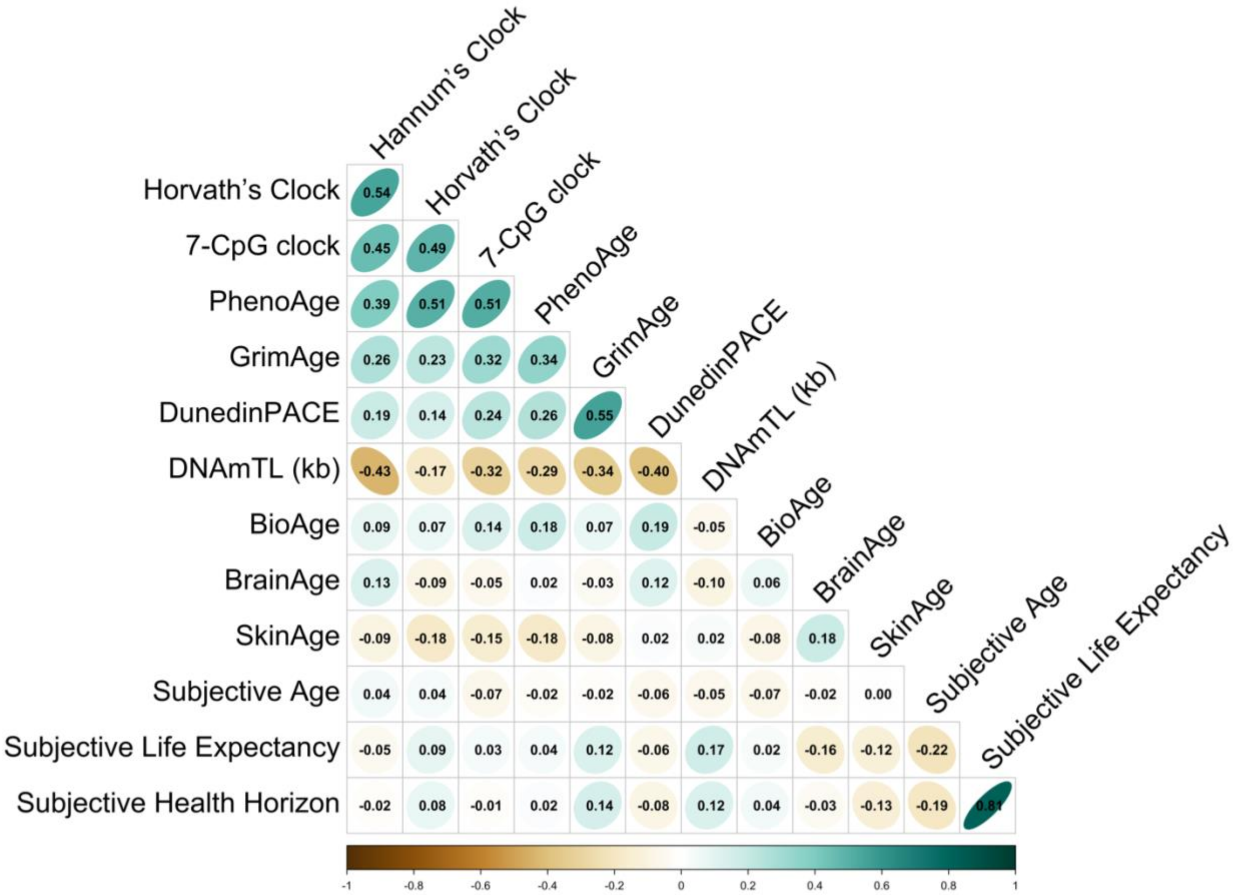

Note. Data for BioAge, BrainAge, and SkinAge taken from baseline assessment. The shape and fill color of the circles indicate the strength of the correlations, with more eccentric ellipses and darker circles representing higher correlations. Positive correlations are represented by green circles and negative correlations are represented by brown circles. Age indicators were scaled with regard to chronological age (exc. DunedinPACE & DNAmTL).

Figure OSM.10. *Intercorrelations among long-term longitudinal changes in the alternative age markers between baseline and follow-up assessments for those who were re-assessed within six years (n = 223).*

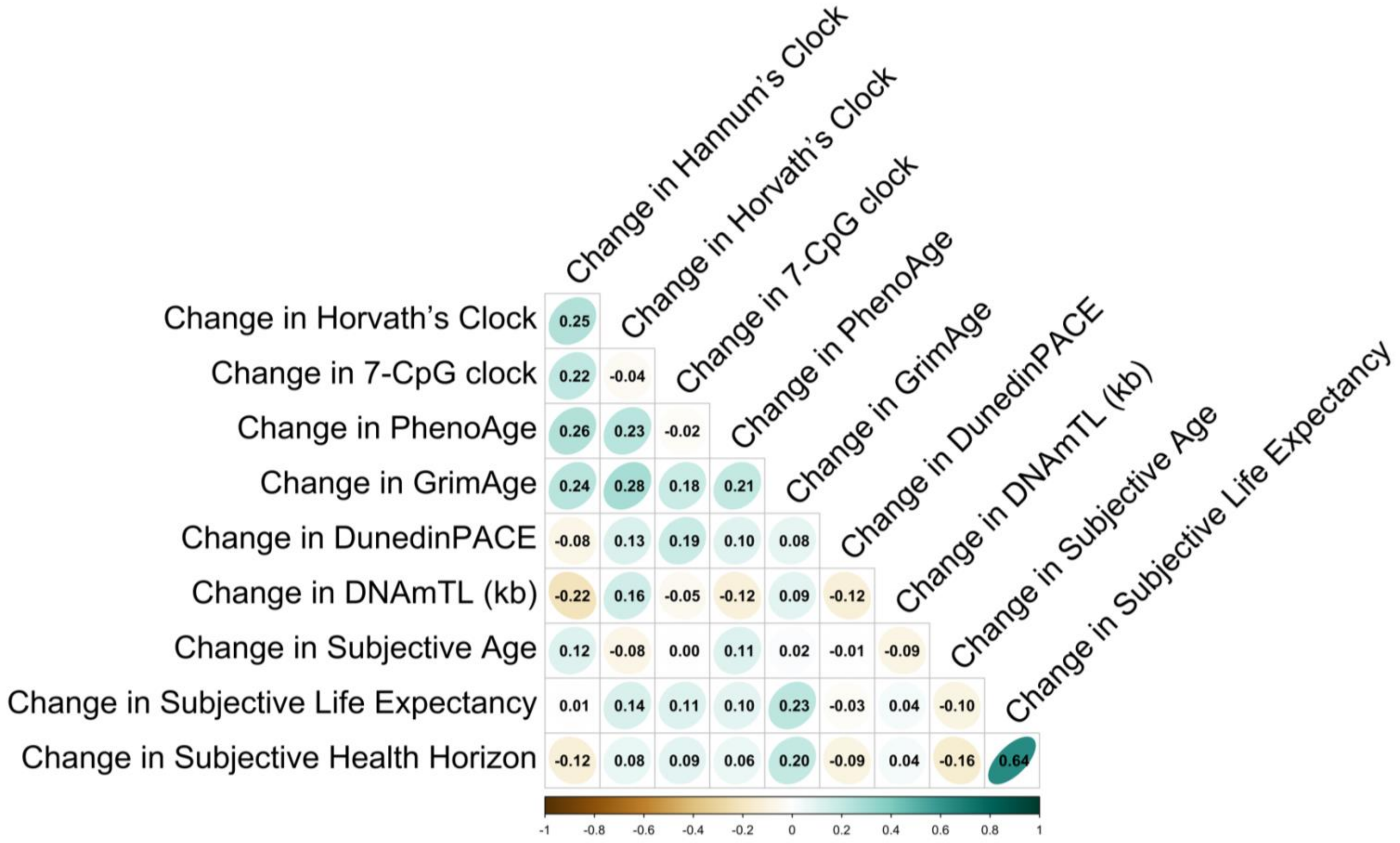

Note. The shape and fill color of the circles indicate the strength of the correlations, with more eccentric ellipses and darker circles representing higher correlations. Positive correlations are represented by green circles and negative correlations are represented by brown circles. Age indicators were scaled with regard to chronological age (exc. DunedinPACE & DNAmTL).

Figure OSM.11. *Associations between subjective time horizon and subjective health horizon indicators.*

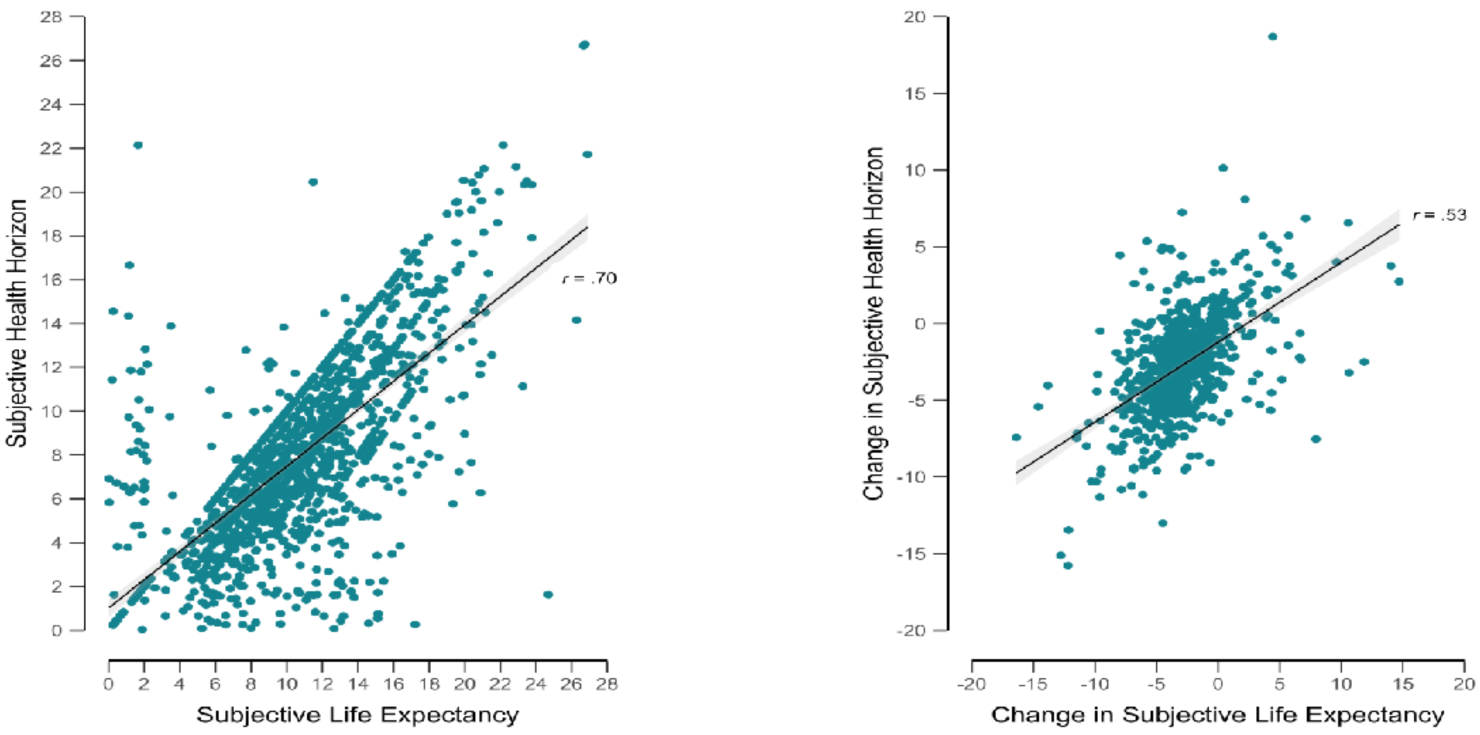

Note. It can be obtained that within a given domain (here, self-reports of subjective time horizon and future health horizon), indicators of aging are moderately interrelated both cross–sectionally and over time.

Figure OSM.12. *Associations between the pace of ageing (DunedinPACE) and skin age (left-hand Panel) and between BioAge and subjective age (right-hand Panel).*

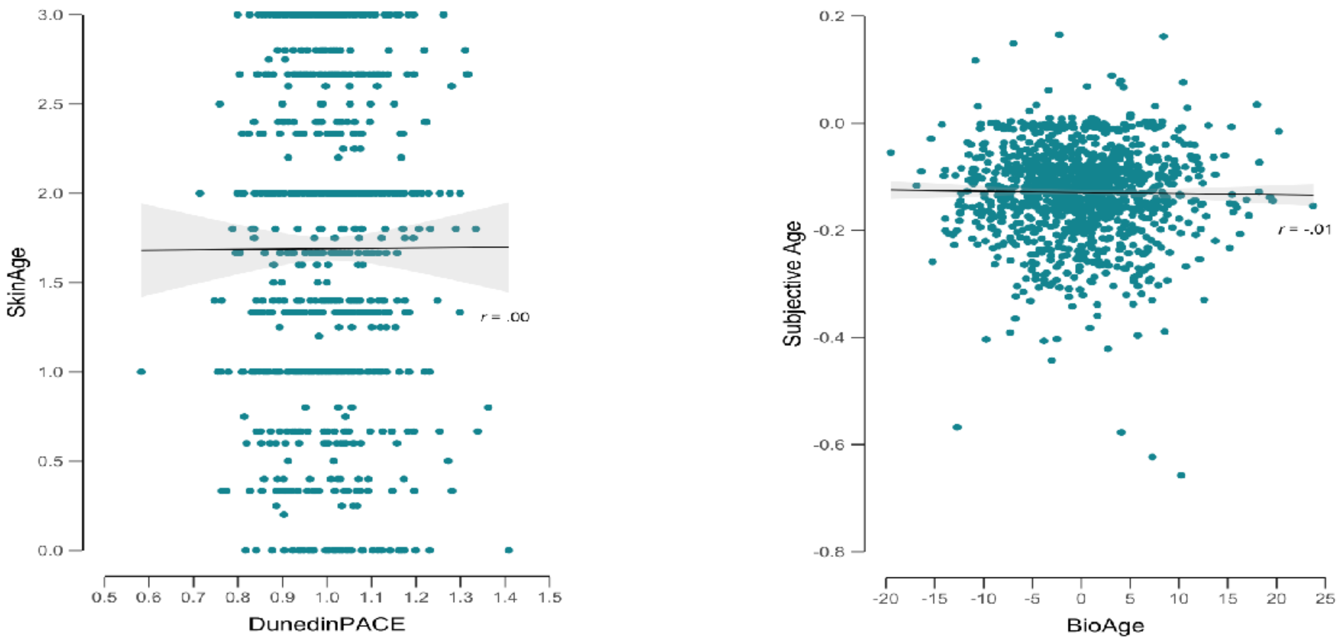

Notes. Across domains, associations of alternative age markers are rare. It can be obtained that other-ratings of older skin age and self–ratings of older subjective age were not associated with the pace of aging (DunedinPACE) and older BioAge, respectively.

Figure OSM.13.

Partial Intercorrelations among the alternative age markers at baseline accounting for women, education, BMI, Smoking

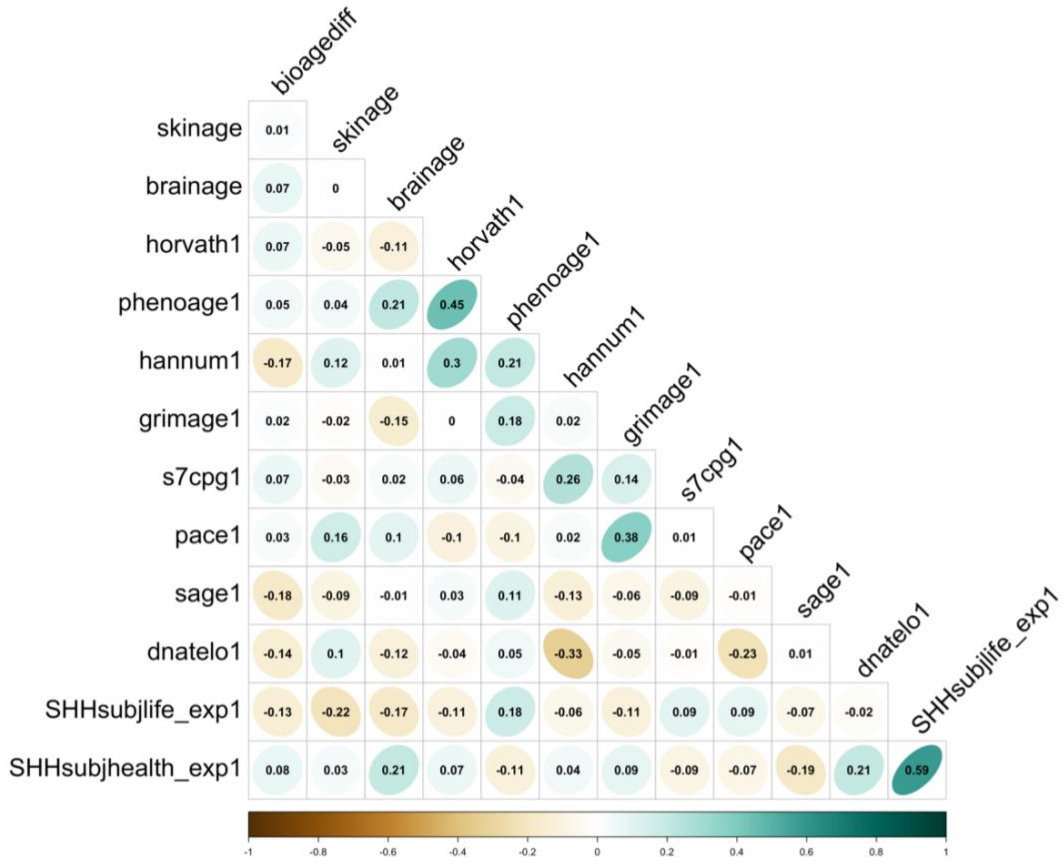

Note. The shape and fill color of the circles indicate the strength of the correlations, with more eccentric ellipses and darker circles representing higher correlations. Positive correlations are represented by green circles and negative correlations are represented by brown circles. Age indicators were scaled with regard to chronological age (exc. DunedinPACE & DNAmTL).

Figure OSM.14.

Partial Intercorrelations among the alternative age markers at follow-up accounting for women, education, BMI, Smoking

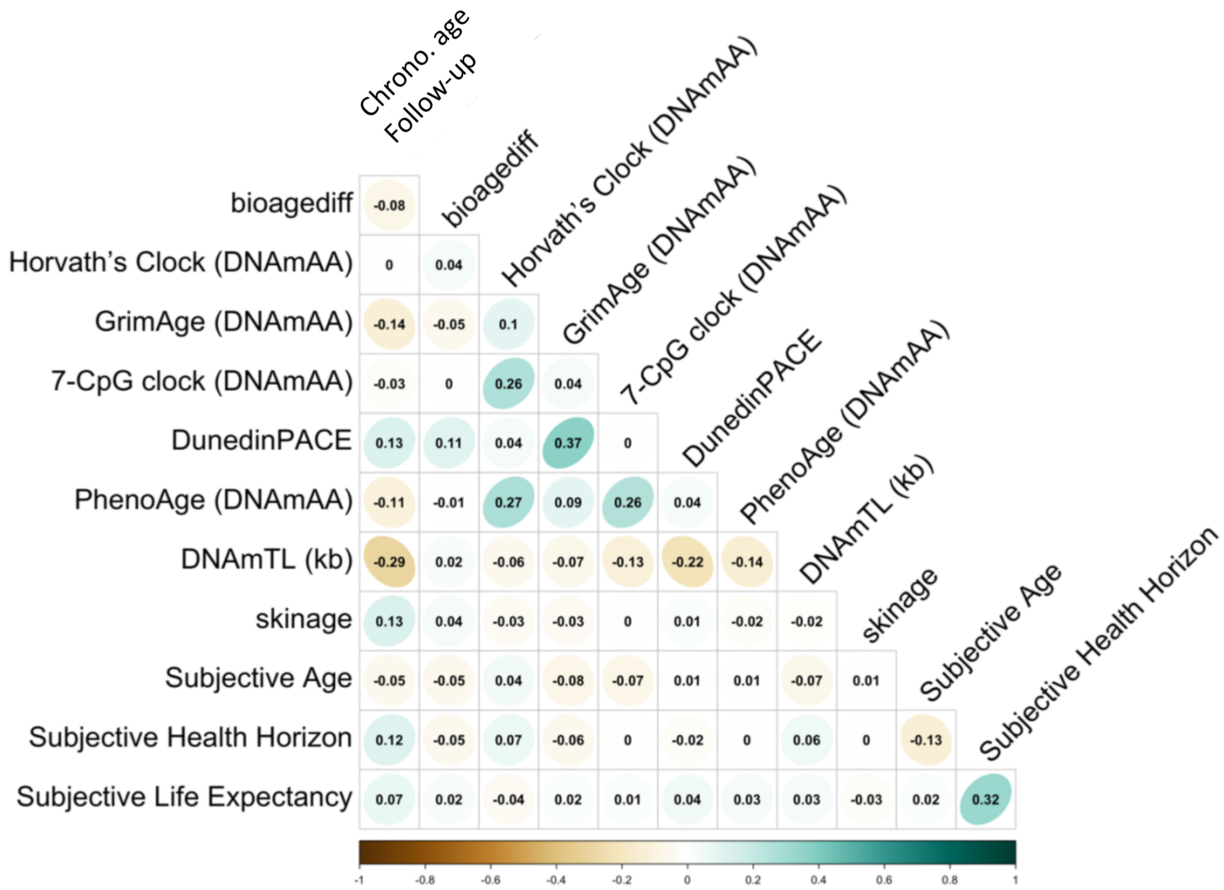

Note. The shape and fill color of the circles indicate the strength of the correlations, with more eccentric ellipses and darker circles representing higher correlations. Positive correlations are represented by green circles and negative correlations are represented by brown circles. Age indicators were scaled with regard to chronological age (exc. DunedinPACE & DNAmTL).

Figure OSM.15.

Partial Intercorrelations among changes in alternative age markers accounting for women, education, BMI, and Smoking.

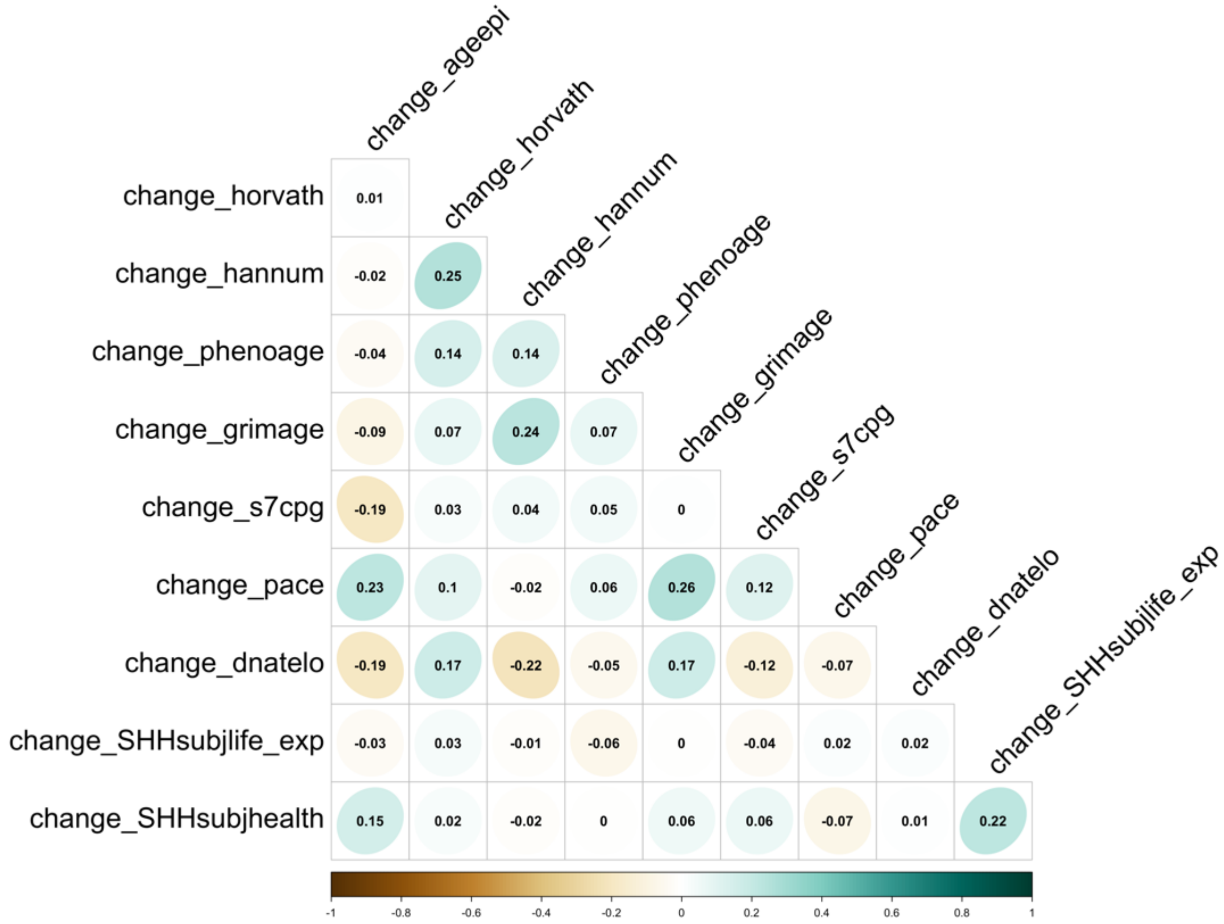

Note. The shape and fill color of the circles indicate the strength of the correlations, with more eccentric ellipses and darker circles representing higher correlations. Positive correlations are represented by green circles and negative correlations are represented by brown circles. Age indicators were scaled with regard to chronological age (exc. DunedinPACE & DNAmTL).

Figure OSM.16.

Associations between Subjective Age and Change in Subjective Age.

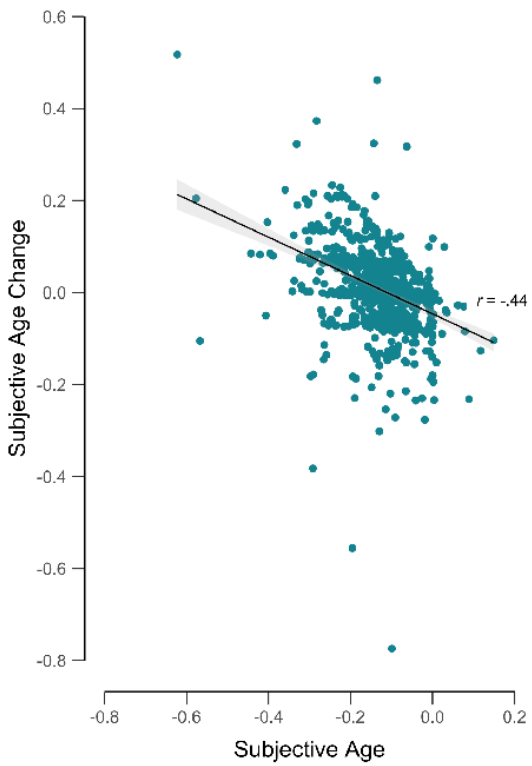

Supplement: glae244_suppl_Supplementary_Material [file glae244_suppl_supplementary_material.pdf]
